# Atomically precise nanoclusters predominantly seed gold nanoparticle syntheses

**DOI:** 10.1038/s41467-023-40016-3

**Published:** 2023-07-21

**Authors:** Liang Qiao, Nia Pollard, Ravithree D. Senanayake, Zhi Yang, Minjung Kim, Arzeena S. Ali, Minh Tam Hoang, Nan Yao, Yimo Han, Rigoberto Hernandez, Andre Z. Clayborne, Matthew R. Jones

**Affiliations:** 1 Department of Chemistry, Rice University, Houston, TX 77005 USA; 2 Department of Chemistry & Biochemistry, George Mason University, Fairfax, VA 22030 USA; 3 Department of Chemistry, Johns Hopkins University, Baltimore, MD 21218 USA; 4 Princeton Materials Institute, Princeton University, Princeton, NJ 08544 USA; 5 Department of Materials Science & Nanoengineering, Rice University, Houston, TX 77005 USA; 6 Department of Chemical and Biomolecular Engineering, Johns Hopkins University, Baltimore, MD 21218 USA; 7 Department of Materials Science and Engineering, Johns Hopkins University, Baltimore, MD 21218 USA; 8 Present Address: Division of Fundamental Research, Petrochemical Research Institute, PetroChina, Beijing, 102206 China

**Keywords:** Nanoparticles, Nanoparticles

## Abstract

Seed-mediated synthesis strategies, in which small gold nanoparticle precursors are added to a growth solution to initiate heterogeneous nucleation, are among the most prevalent, simple, and productive methodologies for generating well-defined colloidal anisotropic nanostructures. However, the size, structure, and chemical properties of the seeds remain poorly understood, which partially explains the lack of mechanistic understanding of many particle growth reactions. Here, we identify the majority component in the seed solution as an atomically precise gold nanocluster, consisting of a 32-atom Au core with 8 halide ligands and 12 neutral ligands constituting a bound ion pair between a halide and the cationic surfactant: Au_32_X_8_[AQA^+^•X^-^]_12_ (X = Cl, Br; AQA = alkyl quaternary ammonium). Ligand exchange is dynamic and versatile, occurring on the order of minutes and allowing for the formation of 48 distinct Au_32_ clusters with AQAX (alkyl quaternary ammonium halide) ligands. Anisotropic nanoparticle syntheses seeded with solutions enriched in Au_32_X_8_[AQA^+^•X^-^]_12_ show narrower size distributions and fewer impurity particle shapes, indicating the importance of this cluster as a precursor to the growth of well-defined nanostructures.

## Abstract (web summary)

In seed-mediated synthesis, small particulate precursors are added to a growth solution to initiate heterogeneous nucleation, allowing access to well-defined colloidal nanostructures. Here, the authors identify that one of the most commonly studied seed solutions is populated by atomically precise gold nanoclusters with 32 atoms.

## Introduction

**Fig. 1:**
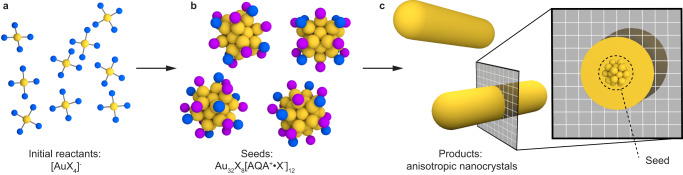
Illustration of a typical anisotropic metal nanoparticle synthesis. Reactions proceed via the rapid reduction of a gold halide salt **a** to nucleate small seed particles **b**, which then act as heterogeneous nucleation sites in a subsequent reaction to facilitate the controlled growth of particles with well-defined shapes **c**. This work identifies the seed intermediates as an atomically precise cluster with 32 gold atoms (yellow), 8 halides (blue), and 12 alkyl quaternary ammonium (AQA)-halide bound ion pairs (purple) as surface ligands.

Anisotropic metal nanoparticles are some of the most ubiquitous and well-studied nanostructures due to their ease of synthesis, high stability, and size- and shape-dependent properties^1^. The use of these structures became commonplace after the development of seed-mediated synthetic strategies in which small nanoparticle “seeds” (Fig. 1a) are added to a separate growth solution (Fig. 1b). Although this approach is the basis for thousands of publications making use of well-defined anisotropic particles, the mechanism is still not fully understood and remains a topic of intense research and debate^2–4^. The size, structure, and surface chemistry of the seeds is of particular concern because downstream particles are presumed to nucleate heterogeneously from them.

The first seed-mediated metal nanoparticle syntheses were developed by Murphy and coworkers and provided enormous advantages by offering particle shape control stemming from the separation of nucleation and growth into distinct steps^5–7^. These reactions typically take place in aqueous solutions of alkyl quaternary ammonium halide surfactants, which bind to particle surfaces and mediate growth^8–10^. Reduction of metal salts and the nucleation of seeds requires the fast injection of powerful borohydride-based reducing agents in order to create small zero-valent metal particles (Fig. 1a). Growth solutions generally contain weaker reducing agents and slower reaction rates to facilitate controlled deposition of metal atoms (Fig. 1b). For example, changing the composition of the growth solution has allowed for the synthesis of dozens of different particle shapes^1^. Although certain mechanistic elements of this approach have been well-established^11^, the role of the seed remains poorly understood^2,3,12^. Given the fact that a vast number of nanosynthesis reactions are predicated, either directly or indirectly, on such seeds^12–20^, it follows that fundamental progress in this domain has been sluggish.

The prevailing hypothesis regarding the structure of seeds synthesized in solutions containing alkyltrimethylammonium halide surfactants is that the majority possess a single-crystalline morphology^2,21,22^. Recently, Vaia, and coworkers^23^ used small-angle X-ray scattering to conclude that many of these seeds are ≈1 nm in size and are therefore better described as nanoclusters rather than particles. However, the identification of any specific Au cluster, its structure, and its surface chemistry has remained elusive. Numerous high-resolution transmission electron microscopy (TEM) studies have investigated the atomic structure of seed particles, but direct imaging of such small nanoclusters is notoriously challenging, as sintering and beam damage make it difficult to correlate images with solution-phase structure^24,25^.

Although ostensibly related, the fields of metal nanoparticle synthesis and solution-phase metal nanocluster chemistry have progressed largely independently. Indeed, advances in the latter field have led to the identification of numerous molecular clusters consisting of well-defined numbers of metal atoms and surface ligands^26,27^. The Brust-Schiffrin method is the most well-known approach for generating gold clusters and typically involves the borohydride-based reduction of metal salts in the presence of relatively strong binding thiol or phosphine ligands^26–30^. Samples are typically characterized by low-fragmentation mass spectrometry techniques^26,28^. While more detailed structural information can be obtained from X-ray crystallography, solved structures are rare because single crystalline samples are difficult to obtain^31,32^. The complexity of the solution phase chemistry has also made it difficult to take advantage of recent advances in the bonding^33^, electronic structure^34^, and catalytic properties of metal clusters^26^ to understand their role in crystal growth^35^. A recent publication^36^ has demonstrated that the structure of a specific Au_56_ cluster shows some similarities to larger triangular gold nanoprisms, including halide-bound {111}-type facets and a prismatic shape, and points to a possible link between the two. However, the lack of internal twin defects in the cluster that are present in the particle and the absence of data showing its ability to seed syntheses leaves its role as a precursor unknown.

**Fig. 2:**
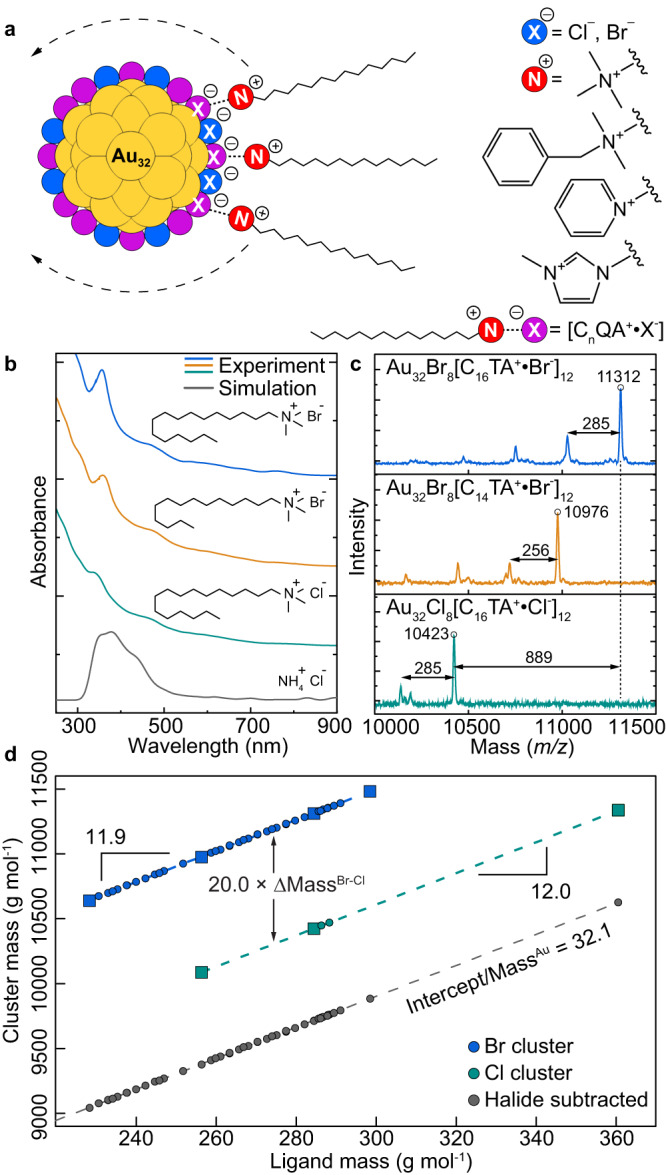
Identification of the chemical formula of gold nanoclusters found in seed solutions. **a** Schematic of the chemical features of Au_32_X_8_[AQA^+^•X^-^]_12_ clusters and the diversity of surface ligands that can stabilize them. **b** Experimental absorption spectra of Au_32_ clusters synthesized with C_16_TAB (blue), C_14_TAB (orange), and C_16_TAC (green) ligands, molecular structures of which are shown as insets. Simulated absorption spectra generated by a combined MD and DFT approach with NH_4_Cl ligands (grey) shows excellent agreement. **c** MALDI mass spectrometry of clusters synthesized with C_16_TAB (blue), C_14_TAB (orange), and C_16_TAC (green) ligands all show an intense peak arising from the intact Au cluster with smaller peaks assigned to the loss of *n* cationic surfactants. **d** Aggregated data showing cluster mass vs. ligand mass for 48 distinct seed syntheses, allowing for determination of the number of surfactant ligands (12), halide anions (20), and gold atoms (32) by linear interpolation/extrapolation. Individual datapoints shown as squares represent clusters with a single type of ligand, circles represent mixed-ligand clusters.

Towards addressing this challenge, here we establish the existence of a 32-atom gold cluster as the major product of the classical AQAX-based seed synthesis developed over two decades ago (Fig. 1). This cluster lacks traditional strongly bound thiol or phosphine ligands, and instead bears 8 halides and 12 halide-cationic surfactant bound ion pairs [AQA^+^•X^-^] as ligands (Fig. 2a). The molecular nature of this cluster allows us to confirm that it possesses all of the properties expected of a “seed” precursor for subsequent particle growth: high reactivity, rapid and promiscuous exchange of surface ligands, and its presence is necessary for the formation of well-defined anisotropic nanostructures. This work establishes a direct link between metal cluster and metal nanoparticle chemistries, and strongly suggests that a full understanding of particle growth mechanisms requires the characterization of processes occurring at the ≈1 nm length scale.

## Results

### Evidence for the molecular nature of “seed” nanoparticles

A typical synthesis of gold seeds involves the fast reduction of an aqueous HAuCl_4_ solution containing hexadecyltrimethylammonium bromide (C_16_TAB) using an aqueous NaBH_4_ solution under rapid stirring^6,7^. This seed synthesis and the Brust-Schiffrin synthesis of Au clusters both use reactions conditions consisting of a gold salt precursor, surface ligand, and rapid NaBH_4_-based reduction^6,7,29^, and therefore the former might have been expected to generate cluster-sized particles as we show in the present work. Indeed, the optical extinction spectra of the as-synthesized seed solution displays sharp, molecule-like absorption features that cannot be attributed to unreacted molecular precursors (Fig. 2b, Supplementary Fig. 1). Although not highlighted, these same optical transitions are visible in the spectra of C_16_TAB-based seeds reported as early as 2003 (Supplementary Fig. 2)^6^. The additional lack of a distinctive plasmon band ca. 505 nm suggests the presence of particles smaller than ≈2 nm, thus lying in the cluster regime^23,37^. This is confirmed via transmission electron microscopy (*vide infra*) and dynamic light scattering measurements showing objects 1-2 nm in size (Supplementary Fig. 3).

To probe which cluster-like species might exist in typical seed solutions, samples were prepared for matrix-assisted laser desorption/ionization (MALDI) time-of-flight mass spectrometry^28^. Interestingly, seed syntheses conducted using related surfactant solutions differing only in the length of the hydrocarbon chain or identity of the halide anion (C_14_TAB, C_16_TAB, and C_16_TAC, hexadecyltrimethylammonium chloride) produce UV-vis spectra with nearly identical absorption features but different mass spectra (Fig. 2b, c, Supplementary Fig. 4). All mass spectra show one intense peak from a large cluster followed by a series of less intense peaks spaced by the mass of the alkyltrimethylammonium cation (256.5 g mol^−1^ for C_14_TA^+^, 284.6 g mol^−1^ for C_16_TA^+^, and 298.6 g mol^−1^ for C_17_TA^+^, Fig. 2c, Supplementary Fig. 4). This indicates the loss of surfactant ligands during the MALDI process and the corresponding gain of protons to keep the core charge constant^38^. Although no other significant peaks are observed in the mass range of 2 – 20 kg mol^−1^ (Supplementary Fig. 4), we cannot rule out the possibility of other AQAX clusters in the sample that might be poorly ionized under our experimental conditions. However, all of the corroborating data (*vide infra*) strongly suggests that a single type of molecular cluster is the dominant species present. Thus, the correspondence in the UV-vis results suggest that each sample contains the same basic cluster core while the differing mass spectra indicates they possess different surface ligands.

Since separately synthesized clusters appear essentially identical except for the molecular composition of their surfactant ligands, differences in their overall mass should indicate the number of ligands composing the cluster. For example, since C_14_TAB and C_16_TAB ligands differ in mass by 28.1 g mol^−1^ and the overall mass of the C_14_TAB- and C_16_TAB-synthesized clusters is 12× this value (335 measured vs. 337 calculated), we conclude that there are 12 alkyltrimethylammonium ligands. Similarly, syntheses containing C_16_TAB and C_16_TAC produce samples with comparable optical absorption features but mass spectra differing by *m/z* of 889 (Fig. 2b, c). Since this value is 20× the difference in mass between chloride and bromide anions (889 measured vs. 889 calculated), we conclude that 20 halides decorate the surface of the clusters. This method of linear interpolation/extrapolation was applied to a library of 48 unique clusters synthesized in the presence of AQAX ligands with different headgroup, side chain, and counterion chemistries (Fig. 2a, Supplementary Figs. 5–7). Separate plots of the cluster mass versus ligand mass for chloride and bromide syntheses each produce linear functions (R^2^ > 0.9999) with slopes and *y*-axis spacing that indicate 12 AQA^+^ cations and 20 X^-^ halide anions, respectively (Fig. 2d). Subtracting the mass of 20 halides from each cluster collapses all points onto a single line (grey points, Fig. 2d), the *y*-intercept of which corresponds to the mass of the inorganic core alone. Assigning this value to Au atoms results in the identification of a 32-atom Au core (see Supplementary Figs. 8, 9). Given the remarkable consistency of these findings across the different cluster species analyzed here, we assign the molecular formula of Au_32_X_8_[AQA^+^•X^-^]_12_, where [AQA^+^•X^-^] indicates a neutral bound ion pair ligand coordinating the cluster surface via the halide^39^. Since these clusters are water-soluble, we conjecture that additional surfactants associate via weak hydrophobic forces to form a charged outer leaflet but do not bind strongly enough to survive the ionization process and therefore do not appear in the mass spectra.

The identification of neutral [AQA^+^•X^-^] bound ion pairs as ligands is without precedent in the cluster literature and rarely discussed in the nanoparticle literature^40,41^. Interestingly, aqueous solutions of the AQAX surfactants used here have been observed to retain 75 – 90% of their halide counteranions in the form of electrostatically-bound ion pairs when they are above their critical micelle concentration^42^. Therefore, the promiscuity with which numerous cationic surfactants form halide-based ion pairs in solution likely explains the large diversity of ligands that can stabilize Au_32_ clusters with minimal change to spectroscopic or structural properties (Supplementary Figs. 5–7)^6,42^.

### Structural analysis of Au_32_X_8_[AQA^+^•X^-^]_12_ nanoclusters

**Fig. 3:**
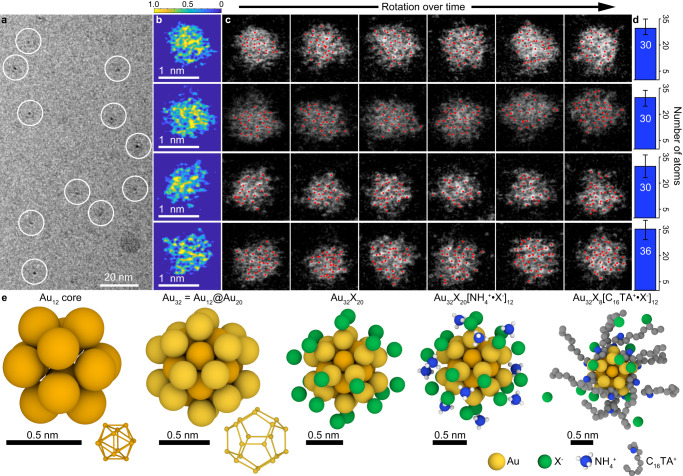
Characterization of the structure of Au_32_X_8_[AQA^+^•X^-^]_12_. **a** Low-resolution cryogenic TEM shows ≈1 nm metal clusters and the absence of larger particles while **b** high-resolution STEM shows clusters with atomic resolution; images in **b** have been processed and represented in false color (see Methods) to visually enhance contrast with greyscale pixel intensities represented by the color scale. **c** Because of beam-induced motion of clusters on a graphene grid, numerous frames of a single cluster can be captured and individual atoms can be identified (red dots), scale is identical to panel **b**. **d** Averaging the Au atom counts over many frames yields values that are consistent with the assignment of a 32-atom core. Error bars represent +/- standard deviation from *N* = 15-18 independent measurements. **e** Proposed pseudo-icosahedral structure of Au_32_X_8_[AQA^+^•X^-^]_12_ built up as concentric radial shells moving left to right: hollow Au_12_ icosahedron, Au_20_ pentagonal dodecahedron, X_20_ halides, (NH_4_)^+^_12_ ammonium headgroups, [C_16_TA^+^•X^-^]_12_ bound ion pairs. Note that the Au_32_X_8_[NH_4_^+^•X^-^]_12_ and Au_32_X_8_[C_16_TA^+^•X^-^]_12_ structures are directly taken from DFT and MD simulation models, respectively.

To corroborate the MALDI results, we used several sample preparation methods to image seeds via cryogenic electron microscopy (cryo-EM) or aberration-corrected annular dark field scanning transmission electron microscopy (ADF-STEM). Flash-frozen seed solutions imaged under cryogenic conditions show numerous ≈1 nm metal particles and the absence of particles 2 nm or larger, consistent with the claim that cluster-sized objects are the predominant seed species (Fig. 3a). Atomically-resolved images of gold clusters of 1-2 nm in size are exceedingly rare, with difficulties being attributed to beam damage and particle agglomeration^24,25^. We observe similar perturbations to clusters when imaging under atomic resolution and therefore cannot unambiguously assign an atomic structure from these data (Fig. 3b). However, ADF-STEM imaging on single-layer graphene supports^43^ preserves the discrete nature of some of the seeds, allowing us to count the number of atoms per particle as they diffuse and rotate during beam irradiation (Fig. 3c, see Methods for details)^44^. A simple image processing algorithm, calibrated to the pixel intensity of single atoms (Supplementary Fig. 10), allows us to quantify the number of gold atoms in each particle for each frame (red dots, Fig. 3c, Supplementary Figs. 11–13). The resulting time-averaged values are in excellent agreement with the mass spectrometry results, indicating an average of ≈32 gold atoms per particle (Fig. 3d, Supplementary Fig. 14).

Notably, the identification of a solution-phase Au_32_ cluster was without literature precedent until two recent publications reported nearly-identical structures determined by X-ray crystallography^45,46^. Both publications show several key properties of the cluster: (1) The inorganic component contains 32 atoms divided into a hollow Au_12_ inner core surrounded by a Au_20_ outer shell; (2) 20 ligand binding sites divided into 12 of one type and 8 of another; and (3) an 8^+^ charge on the gold core. Our present results indicate a nearly identical set of properties. For example, we observe a 32-atom Au cluster bearing 20 total ligands that are divisible into 12 neutral [AQA^+^•X^-^] ligands and 8 negatively charged halide ligands requiring an 8^+^ charge on the gold core for overall charge neutrality. The numerical elements of this cluster (8, 12, and 20) are relevant, as both structures identified by X-ray data assign pseudo-icosahedral symmetry in which the 20 faces are split into distinguishable sets of 8 and 12 represented by the different ligands^45,46^. In addition, the earlier publications noted that changes to the ligand identity render the UV-vis spectra and core structure largely unchanged, as we have observed here^46^.

To determine if the previously-reported icosahedral Au_32_ structure is consistent with our results, we performed a series of simulations to predict the cluster’s optical properties and compared them to our experimental results. The AQAX ligand shell was treated with classical molecular dynamics (MD) assuming a rigid Au core (Supplementary Fig. 15). After equilibration, randomly selected structures, with the ligands truncated to NH_4_^+^ and X^-^, were used as inputs for time-dependent density functional theory (TD-DFT) calculations to generate a distribution of optical spectra (see Supplementary Fig. 16). Previous work on Au_32_ suggested the importance of the steric bulk of the surface ligands in providing stabilization of the structure^46^. The combination of the MD and TD-DFT treatments of the ligands and core allows us to capture this effect, presumably resulting from the packing of AQAX ligands around the particle surface. The simulated and experimental spectra show excellent agreement (Fig. 2b, Supplementary Fig. 17), with several prominent transitions appearing in the experimental data (at wavelengths of 806, 639, and 381 nm with a shoulder at 513 nm) also appearing in the simulated data (at wavelengths of 831, 699, and 386 nm with a shoulder at 436 nm). Additionally, imaging of dried seed samples in TEM mode with a low electron dose rate allowed us to capture numerous examples of clusters oriented to the same fundamental 2-, 3-, and 5-fold symmetry axes as the proposed structure (Supplementary Fig. 18)^45,46^. Taken together, these results strongly suggest that Au_32_X_8_[AQA^+^•X^-^]_12_ adopts a hollow pseudo-icosahedral structure.

### Ligand exchange in Au_32_X_8_[AQA^+^•X^-^]_12_

**Fig. 4:**
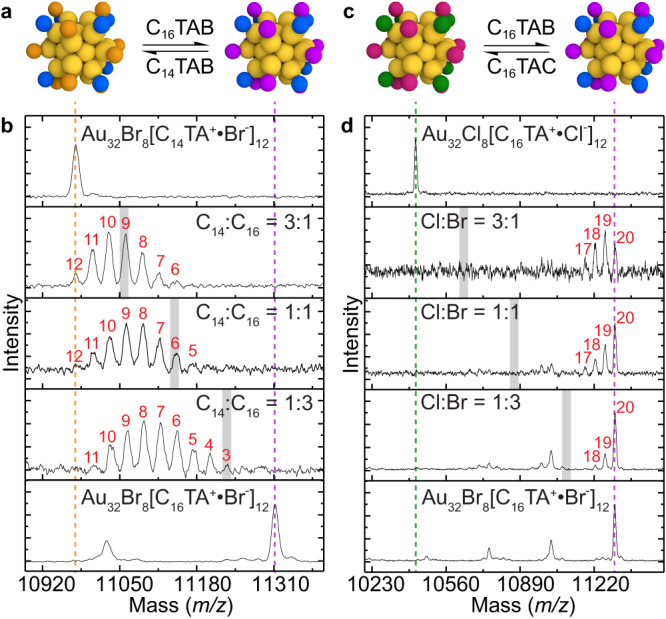
Mass spectrometry analysis of nanocluster ligand exchange. **a, b** Exchange of the bound ion pair and **c, d** halide ligands on Au_32_X_8_[AQA^+^•X^-^]_12_ clusters can both be resolved in MALDI spectra. **b, d** Dotted lines indicate the mass of single-ligand clusters and grey bars indicate the location of the distribution of mixed-ligand clusters if binding of each molecule was equally favorable. Peaks are indexed in red to denote the number of exchanged ligands and the ratio of molecules in solution are given as insets. **a, b** Equilibration to a mixture of ligands with differing alkyl chain lengths results in a preference for [C_14_TA^+^•Br^-^] over [C_16_TA^+^•Br^-^] bound ion pairs. **c, d** Equilibration to a mixture of ligands with differing halide counterions results in a preference for Br over Cl. (Blue circles for Br^-^; green Cl^-^; orange [C_14_TA^+^•Br^-^]; magenta [C_16_TA^+^•Br^-^]; red [C_16_TA^+^•Cl^−^]).

Implicit in the idea that Au_32_ clusters are mechanistically relevant in nanoparticle synthesis is the requirement for the surface ligands to be sufficiently labile so as to exchange with new ligands in solution and/or allow for the deposition of reduced metal atoms. To probe this, Au_32_Br_8_[C_16_TA^+^•Br^-^]_12_ samples were exposed to solutions containing a mixture of C_16_TAB and C_14_TAB in differing ratios (Fig. 4a). Mass spectrometry indicates the formation of a distribution of mixed-ligand clusters of the form Au_32_Br_8_[C_14_TA^+^•Br^-^]_*x*_[C_16_TA^+^•Br^-^]_12-*x*_ (Fig. 4b). Similarly, when Au_32_Cl_8_[C_16_TA^+^•Cl^-^]_12_ samples were exposed to solutions containing a mixture of C_16_TAB and C_16_TAC of differing ratios (Fig. 4c), clusters consisting of mixed-halide ligands of the form Au_32_Br_*y*_Cl_8-*y*_[C_16_TA^+^•Br^-^]_*x*_[C_16_TA^+^•Cl^-^]_12-*x*_ were observed (Fig. 4d). We find that for both the halide and bound ion pair exchange reactions, the new distributions of mixed-ligand clusters are reached in 10-15 min, or nearly as quickly as samples can be prepared for analysis (Supplementary Figs. 19, 20). In addition, the same distribution of ligands is observed regardless of which single-ligand cluster is added to the mixed-surfactant solution, indicating a fully equilibrated state of ligand exchange (Supplementary Fig. 21). Au_32_X_8_[AQA^+^•X^-^]_12_ clusters must therefore possess a highly dynamic ligand shell that is quite unlike previously-reported Au clusters strongly-bound by thiol or phosphine ligands^26,28^. This result is consistent with the overall finding of this work that these clusters act as seeds in nanoparticle synthesis.

The molecular nature of the Au_32_ cluster allows for quantitative measurement of chemical properties that would be difficult, if not impossible, to determine if seed solutions contained nanoparticles with range of different sizes. For example, in all cases, the center of the distribution of mixed-ligand clusters is biased relative to the ratio of ligands in solution (grey bars, Fig. 4b, d), indicating a binding preference for some ligands over others. The ratio of the binding constants of the ligands to the cluster can be estimated from a simple two-component Langmuir isotherm model (see Methods). The ligand ratio can be obtained from the center of the mixed-ligand cluster distributions (Supplementary Fig. 22). This analysis indicates that [C_14_TA^+^•Br^-^] binds to Au_32_ 5.5-fold more strongly than [C_16_TA^+^•Br^-^], which is surprising given the ubiquity of C_16_TAB as a surface ligand in these systems (Fig. 4b, Supplementary Fig. 23). As expected, Br counterions bind 24-fold more strongly that Cl, which is consistent with numerous studies on halide binding to metal surfaces (Fig. 4d, Supplementary Fig. 23)^11^. Such results allow for nanoparticle growth reactions to be understood at a greater level of mechanistic detail, as different seed chemistries can be compared quantitatively and correlated to different synthetic outcomes.

### The importance of Au_32_ clusters in nanoparticle synthesis

**Fig. 5:**
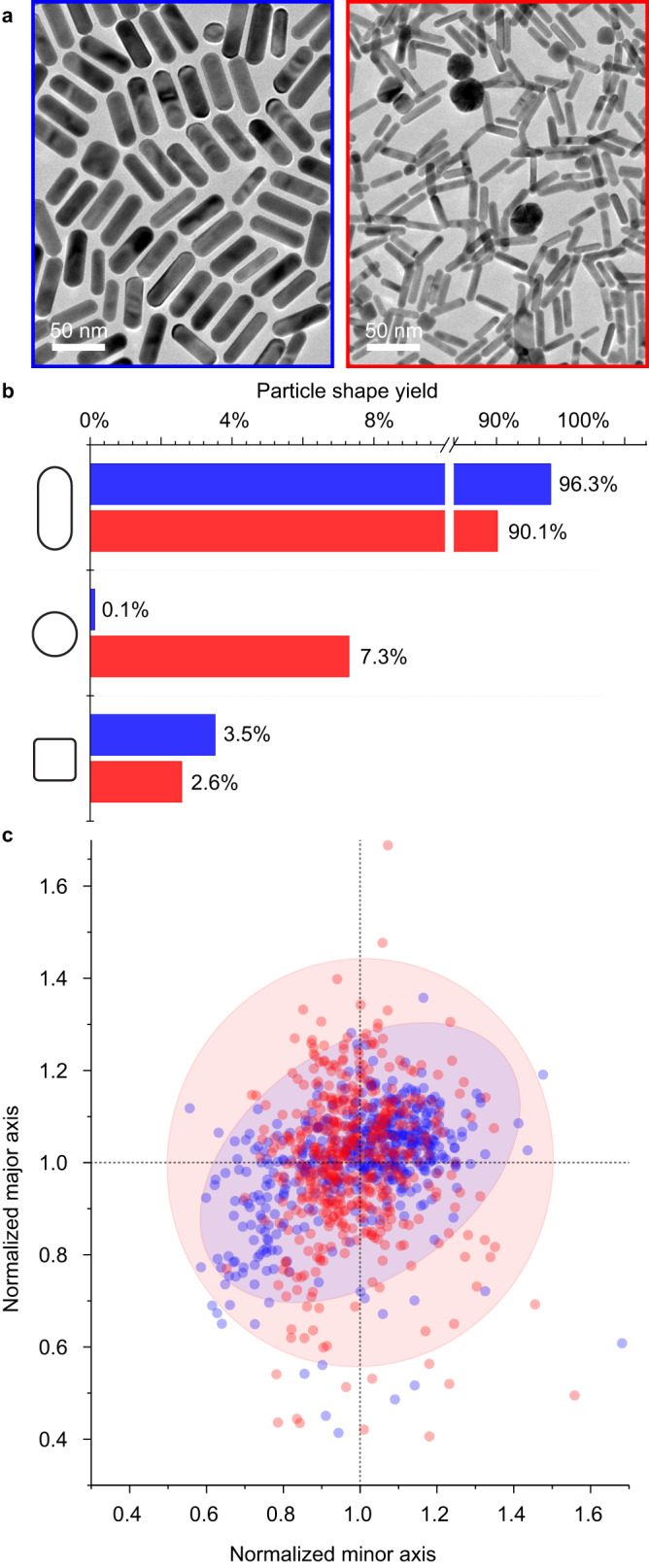
Morphological analysis of gold nanorods synthesized with traditional seed solutions (red) or solutions enriched in Au_32_ nanoclusters (blue). **a** Representative TEM images. **b** Percent of total particles constituting rod, sphere, and cube shapes. **c** Size distribution of gold nanorod major and minor axes normalized to the average with shaded region representing 95% confidence ellipse. Full datasets (see Supplementary Fig. 24b) consist of *N* = 766 and 2,990 measurements for Au_32_ and traditional seeds, respectively. 500 points from each dataset have been selected at random to improve visibility.

To determine what mechanistic relevance Au_32_X_8_[AQA^+^•X^-^]_12_ might have for guiding nanoparticle growth, we compared the quality of gold nanorod syntheses seeded from solutions enriched with a higher population of Au_32_ (blue data points, Fig. 5) to those seeded from solutions created according to the original reports (red data points, Fig. 5)^6,7^. Importantly, nanorods synthesized using Au_32_-enriched seeds showed fewer impurity shapes (4% vs. 10%, Fig. 5b) and a narrower size distribution along their major and minor axes (shaded ellipses are 95% confident intervals, Fig. 5c) from their traditional seed counterparts. This data was collected via an image processing algorithm that allowed us to measure over 40,000 different particles positioned randomly on the TEM grid (see Methods, Supplementary Fig. 24), thus giving a high degree of confidence in the statistical significance of the finding. Repeating the comparison of Au_32_ and traditional seed-synthesized nanorods under identical conditions five separate times shows similar results of improved yield and uniformity, thus highlighting the robustness of this observation (Supplementary Fig. 25). Interestingly, in all cases, Au_32_ seeds produce rods that are larger than those generated by traditional seeds (Fig. 5) which implies that there is not a one-to-one correspondence between nanocluster seeds and final nanoparticle products; it is the subject of future work to map the complex space of such intercluster reaction pathways. Regardless, from these data we can conclude that Au_32_X_8_[AQA^+^•X^-^]_12_ is indeed playing an important role in the synthesis of larger nanoparticle products.

## Discussion

Our findings have several significant implications and limitations. First, while the seed solutions synthesized for the past 20 years have been presumed to contain nanoparticles, we now show that the predominant entity is Au_32_X_8_[AQA^+^•X^-^]_12_, which is an inorganic molecular cluster. This finding provides a pathway to deeper understanding of growth reactions than what was available previously by clarifying the mechanistic starting point for nanoparticle synthesis. However, it is still unclear if Au_32_X_8_[AQA^+^•X^-^]_12_ is the only cluster capable of seeding growth or if an entire class of AQAX-based clusters with different sizes/shapes may appear under slightly different seed syntheses, leading to different particle outcomes. Second, we observe that Au_32_X_8_[AQA^+^•X^-^]_12_ clusters are stable for a week at most, at which point they will have undergone a coalescence process to become larger spherical gold particles >2 nm in size because of their high surface energy^47^. This implies that cluster-cluster fusion is a facile process and several of the “seed aging” protocols that can be found in nanoparticle syntheses^23,48,49^ may be acting to redistribute the cluster population, or even facilitate the size-focusing that has been observed in other gold nanocluster systems^50^. Third, the highly-symmetric pseudo-icosahedral structure of Au_32_X_8_[AQA^+^•X^-^]_12_ provides little insight into how and why lower-symmetry nanoparticle products (e.g., rods or tetrahedra) arise in a highly symmetric crystal system such as FCC^51^. However, the rapid ligand dynamics and cluster coalescence we observe suggests that solution-phase inter-cluster reaction chemistry may be the relevant length scale at which symmetry breaking and other important mechanistic processes occur. Overall, these results point the field in several new directions, toward a science of nanoparticle synthesis that is predicated on atomic precision and deeper mechanistic understanding.

## Methods

### Chemicals

HAuCl_4_∙3H_2_O (≥99.9% trace metal basis), NaBH_4_ (≥99.99% trace metal basis), NaI (≥99.999% trace metal basis) dodecyltrimethylammonium bromide (C_12_TAB, BioXtra, ≈99%), tetradecyltrimethylammonium chloride (C_14_TAC, TCI, >98.0%), tetradecyltrimethylammonium bromide (C_14_TAB, >99%), hexadecyltrimethylammonium chloride (C_16_TAC, ≥98%), hexadecyltrimethylammonium bromide (C_16_TAB, TCI, >98%), heptadecyltrimethylammonium bromide (C_17_TAB, TCI, >98%), cetylpyridinium bromide (CPB, TCI, >96%), cetylpyridinium chloride (CPC, TCI, >98%), benzylcetyldimethylammonium chloride (BDAC, n/a %), 1-hexadecyl-3-methylimidazolium chloride (HMIC, Toronto research chemicals, n/a %), trans-2-[3-(4-tert-Butylphenyl)−2-methyl-2-propenylidene]malononitrile (DCTB, ≥98%), [[3,5-bis(1,1-dimethylethyl)−4-hydroxyphenyl]methylene]propanedinitrile (Tyrphostin A9, TA9, malonoben, TCI, >98%), trifluoroacetic acid (TFA, 99%), diammonium hydrogen citrate (DHC, ammonium citrate dibasic, ≈98%), L-ascorbic acid (AA, BioXtra, ≥99.0%). All chemicals were obtained from Sigma-Aldrich unless otherwise stated and were used as received.

### Synthesis of Au_32_ nanoclusters

The Au_32_ cluster synthesis is derived from the standard seed synthesis reported by El-Sayed and coworkers^6^. First, 0.625 mL of 10 mM HAuCl_4_•3H_2_O solution and 6.25 mL of 0.2 M C_16_TAB solution are added to 16.63 mL H_2_O. Next, 1.5 mL of ice-cold 10 mM NaBH_4_ solution is quickly injected into the above solution under vigorous stirring and kept under stirring for 2 min. The C_16_TAB solution is replaced with other surfactant solutions at comparable concentrations to produce clusters with different ligands.

### Synthesis of traditional nanoparticle seed solution

The typical NP seed solution is synthesized per the method developed by El-Sayed and co-workers^6^. First, 5.0 mL of 0.5 mM HAuCl_4_•3H_2_O solution and 5.0 mL of 0.2 M C_16_TAB solution. Next, 0.60 mL of ice-cold 10 mM NaBH_4_ solution is quickly injected into the above solution under vigorous stirring and kept under stirring for 2 min.

### Synthesis of AuNRs

The AuNR syntheses are based on the method developed by El-Sayed and co-workers^6^. 0.5 mL 10 mM HAuCl_4_ solution, 5 mL 200 mM C_16_TAB solution, 5 mL H_2_O, 90 μL 10 mM AgNO_3_ solution, and 57 μL 100 mM AA solution are mixed under vigorous stirring; then 50 μL, 75 μL, 100 μL, or 200 μL typical NP seed solution or Au_32_ seed solution is quickly injected to initiate the growth of AuNRs. Note that the total volume of the solution is kept consistent by adjusting the volume of H_2_O across these samples, 50 μL seed solution corresponds to 5 mL H_2_O; 75 μL seed 4.975 mL H_2_O; 100 μL seed 4.950 mL H_2_O; 200 μL seed 4.850 mL H_2_O. The solution is kept still at 28 °C for 2 h to complete the growth of AuNRs. The resultant nanoparticles including gold nanorods, nanospheres, and nanocubes are centrifuged at 21,000x *g* for 30 min; the precipitate is collected by decanting the supernatant and then dissolved in water for TEM sample preparation.

### UV-Vis-NIR absorption

A Cary 5000 spectrometer was used to acquire absorption spectra at 200-1000 nm range with a baseline of water.

### MALDI-ToF-MS

MALDI matrices, DCTB and TA9 were dissolved in a solution of 50:50 (v/v) acetonitrile: H_2_O with 0.1 vol% TFA and 0.5 − 1 mg mL^−1^ DHC. 2 μL matrix solution was dried on the ground steel target plate, and 2 μL cluster solution was dried on top of the matrix thin layer. A Bruker MALDI-ToF-MS instrument equipped with nitrogen laser (337 nm) was used to acquire the spectrum at 5000-20,000 range in the reflectron mode. The acquired spectrum was then baseline-subtracted, smoothened, and labeled in the Bruker flexanalysis software.

### Electron microscopy

Cryo-EM imaging of Au_32_X_8_[AQA^+^•X^-^]_12_ nanoclusters was collected on a 300 kV Cs-corrected Titan Krios using a K2 Summit detector (with GIF Bio-Quantum Energy Filters, Gatan). We collected the raw movies in K2 counted mode at a magnification of 215,000x (in energy-filtered TEM [EFTEM] mode, spot size 6, C2 aperture 70 μm) with a pixel size of 0.536 Å. The total exposure time was set to 2.4 s with a 0.075-s frame time to generate 32-frame gain normalized stacks in MRC format. The total dose for a stack is 49 e^2^ Å^−2^.

STEM imaging of Au_32_X_8_[AQA^+^•X^-^]_12_ nanoclusters was collected on a 300 kV Titan Themis with a ≈10 pA probe current. We took stack images with 20 nm field of view and ≈1 s acquisition time per frame. A 30 mrad convergence angle and an approximately 40 mrad inner collection angle were used for all ADF-STEM images, whose contrast is proportional to Z^γ^, where Z is the atomic number and 1.3 < γ < 2.

For HRTEM imaging of Au_32_X_8_[AQA^+^•X^-^]_12_ nanoclusters, the as-synthesized solution was cooled at 0 °C for 30 min and centrifuged at 4 °C for 30 min before being spotted on the grid. Copper TEM grids with ultrathin carbon film from Ted Pella was treated with chloroform to remove the formvar layer. 10 μL sample was deposited on the carbon side of the grid. HRTEM images were acquired on a JEOL JEM 2100 F TEM operating at 200 kV.

TEM imaging of AuNRs was accomplished by depositing 10 μL of sample on the carbon side of the regular 400 mesh TEM grids. Images were acquired on a JEOL JEM 2100 F TEM operating at 200 kV.

### Size analysis of AuNRs

The automated morphological analysis of the AuNR samples are performed via the processing of TEM images with a MATLAB script modified from Mirkin and co-workers^52^. The aspect ratio threshold is set as 1.3; the pixel conversion factor 0.4274 nm per pixel; the solidity threshold 0.8; the intensity threshold 70. Pixel thresholds were set to 50, 100, and 400 for images collected at 10,000x, 25,000x, and 50,000x magnification, respectively. A total of ≈40,000 particles were outlined, measured, and categorized for 3 different seed volumes (50, 75, 200 μL) and both seed types (Au_32_ nanoclusters and traditional El-Sayed^6^) to generate the size distributions shown in Supplementary Figs. 24 and 25. To generate the plots for Fig. 5, 500 points were selected at random from each dataset to aid in the visualization of the data.

### Image processing

All micrographs are presented without modification except those in Fig. 3b, which were processed as follows: application of a gaussian filter with a standard deviation of 0.75, subtraction of all pixels by the average pixel value, normalization, saturation of the top 1% and bottom 40% of non-zero pixel values, and colored with the Parula colormap. The colorscale values in Fig. 3b represent the greyscale pixel intensities of the processed images.

### Computational methods

The starting structure for the Au_32_Cl_8_[C_16_TA^+^•Cl^-^]_12_ and Au_32_Br_8_[C_16_TA^+^•Br^-^]_12_ cluster was taken from the previously reported Au_32_Cl_8_(R_3_P)_12_ structure^46^. The PR_3_ groups were replaced with [C_16_TA^+^•Cl^-^] groups and eight additional Cl^-^ groups were added to the structure. To obtain the Au_32_Br_8_[C_16_TA^+^•Br^-^]_12_ structure all Cl atoms were replaced with Br atoms.

To gain insight into the possible effects of the full C_16_TA^+^ ligand, classical molecular dynamic simulations were performed using the Nanoscale Molecular Dynamics program, version 2.13b1 (NAMD 2.13b1)^53^. The Au_32_ cluster was constructed with three layers of C_16_TABs. The first layer was packed with 12 C_16_TABs resulting in a Au_32_Br_8_[C_16_TA^+^•Br^-^]_12_ model structure. We packed a second layer of C_16_TABs (26 C_16_TAB) around Au_32_Br_8_[C_16_TA^+^•Br^-^]_12_ under two scenarios: with and without first equilibrating the underlying Au_32_Br_8_[C_16_TA^+^•Br^-^]_12_ structure. The second layer of C_16_TABs (unbound inner C_16_TABs) were packed around within a radius of 6.8–8.9 Å keeping the hydrophilic N^+^ head pointing towards cluster core. A third layer of C_16_TABs (200 C_16_TABs) was packed around each of the equilibrated two-layer structures within a radius of ≈12 Å keeping their N^+^ headgroups pointing towards the water solvent. Second and third layers of C_16_TABs were packed using Packmol^54^. Au atoms and the eight Br atoms that are covalently bound to the Au core were fixed during the molecular dynamics simulations due to the unavailability of a gold forcefield that can properly stabilize the Au_32_ geometry under the assumption that the Au_32_ structure is relatively rigid. The all-atom CHARMM27 force field and its parameters were used to model all interactions^55^. The mixing rules for Br Lennard-Jones interactions^56^ and additional terms to account for Au polarization^57^ were used. The CTA^+^ ligand topology and parameters were obtained from CHARMM-GUI. A representative stepwise equilibrated structure with C_16_TABs, 12 bound inner 26 unbound inner 200 outer, is shown in Supplementary Fig. 15. It was found that 26 C_16_TABs can be packed in the second layer while keeping the hydrophilic N^+^ head pointing towards the gold cluster. 200 CTABs in the third layer were sufficient to obtain a spherical vesicle-type structure. After equilibration, a snapshot from the simulation was obtained. The second and the third layers were removed and the alkyl carbon chain in C_16_TABs truncated to serve as inputs for the DFT calculations.

### Reporting summary

Further information on research design is available in the Nature Portfolio Reporting Summary linked to this article.

## Supplementary information

Supplementary Info

Reporting Summary

## Data Availability

Electron microscopy and mass spectrometry datasets generated during and/or analyzed during the current study are available in the Supplementary Information file. All data that support the findings of this study are available from the corresponding author upon request.

## Code availability

The MATLAB code used to find and categorize atoms from ADF-STEM images is available from the corresponding author upon request.
