# Supplementary material for: Atomically precise nanoclusters predominantly seed gold nanoparticle syntheses
Source: Nat Commun. 2023 Jul 21;14:4408. doi: 10.1038/s41467-023-40016-3 (PMC10362052; doi:10.1038/s41467-023-40016-3)
Supplement: Supplementary file 1 — Supplementary Info [file 41467_2023_40016_MOESM1_ESM.docx]

Supplementary Information

**Atomically-Precise Nanoclusters Predominantly Seed Gold Nanoparticle Syntheses**

**AUTHOR LIST:**

Liang Qiao,^1,8^ Nia Pollard,^2^ Ravithree D. Senanayake,^3^ Zhi Yang,^1^ Minjung Kim,^1^ Arzeena S. Ali,^1^ Minh Tam Hoang,^3^ Nan Yao,^4^ Yimo Han,^5^ Rigoberto Hernandez,^3,6,7^ Andre Z. Clayborne,^2^ and Matthew R. Jones^1, 5,^*

**AFFILIATIONS:**

^1^Department of Chemistry, Rice University, Houston, Texas 77005

^2^Department of Chemistry & Biochemistry, George Mason University, Fairfax, Virginia 22030

^3^Department of Chemistry, Johns Hopkins University, Baltimore, Maryland 21218

^4^Princeton Materials Institute, Princeton University, Princeton, NJ 08544

^5^Department of Materials Science & Nanoengineering, Rice University, Houston, Texas 77005

^6^ Department of Chemical and Biomolecular Engineering, Johns Hopkins University, Baltimore Maryland 21218

^7^Department of Materials Science and Engineering, Johns Hopkins University, Baltimore, MD 21218

^8^Present Address: Division of Fundamental Research, Petrochemical Research Institute, PetroChina, Beijing 102206, China.

*To whom correspondence should be addressed: mrj@rice.edu

**SUPPLEMENTARY METHODS AND DISCUSSION**

**Single Atom Counting Image Processing**

Atoms are identified with a local maximum identification process. To calibrate the algorithm, single atoms in the field of view are used as references for setting the image processing parameters. Statistical results of the number of atoms in each cluster are then derived from the localization outcomes for each image frame.

Processing protocol for one image stack

1. Crop out single atoms in the first frame
2. Process the single atom images to calculate $\text{Area}_{\text{Au}}$ (pixel area of single Au atoms) and $\text{I}_{\text{Au}}$ (pixel intensity of single Au atoms). Based on $\text{Area}_{\text{Au}}$ and $\text{σ}_{\text{Area}_{\text{Au}}}$ (standard deviation of single Au atoms pixel areas for each Au atom in the same image frame), we obtain the $\text{KS}_{\text{Au}}$ (Kernel size of the maximum filters).
3. Identify atoms in the clusters using a maximum localization algorithm, with kernel size of $\text{KS}_{\text{Au}}$, remove local maximum pixels with pixel intensities lower than $\text{I}_{\text{Au}}$.
4. Calculate the number of atoms in the cluster within the same image stack.

For each single atom cropped from frame 0, we threshold background pixels and calculate the number of pixels in the atom (excluding the background pixels), the pixel intensity in the single atom image, and sum up the intensities of the pixels in the single atom. Based on the composition of the Au_32_ cluster, the major elements seen in STEM images should be Au and Br atoms, and thus 2 types of atoms should be found in the cropped single atom images. To mathematically determine if there are two discrete groups of single atom pixel intensities, a K-means clustering algorithm (sklearn.cluster.kmeans package) was used with n_clusters=2. A clear boundary between two types of atoms is seen when the 2D integral (sum of pixel intensities) of the single atoms is used as the metric for atom classification (Supplementary Table 1). If these two classes of 2D integral values correspond to single Au or Br atoms, the ratio of their average intensities raised to an exponent γ should correspond to the ratio of their atomic number raised to the same exponent γ, where γ = 1.7 has been determined empirically,^1-3^ i.e. (Z_Au_/Z_Br_)^1.7^ = 3.98.^4^ The calculated value of 3.13 is in good agreement given the small number of measured single atoms and is consistent with the hypothesis that single Au and Br atoms are the predominant species and separately identifiable in ADF-STEM images. Single atoms in class 1 were therefore classified as Au atoms and their measured parameters used in the localization algorithm.

**Supplementary Table 1.** 2D integrals of single atoms in ADF-STEM images can be classified into 2 groups.

| Image stack | 1-1 | 1-2 | 1-3 | 1-4 | 1-5 | 2-2 |
| --- | --- | --- | --- | --- | --- | --- |
| class 1  2D integral | 11687 ±1295 | 9687±1486 | 6483±1260 | 10228±1416 | 10458±1135 | 9061±1610 |
| class 2  2Dintegral | 5466±1566 | 5244±1096 | 3295±521 | 5171±1542 | 5415±1402 | 4837±909 |

The atom localization algorithm first applies a maximum filter to each frame in the image stack using a kernel size of $\text{KS}_{\text{Au}}$ (scipy.ndimage.maximum_filter) where:

$\text{KS}_{\text{Au}} = \sqrt{\bar{\text{Area}_{\text{Au}}}\text{- }\text{σ}_{\text{Area}_{\text{Au}}}}$ (Supplementary eq. 1)

and $\text{σ}_{\text{Area}_{\text{Au}}}$ refers to the standard deviation of the pixel area and is subtracted from $\text{Area}_{\text{Au}}$ in order to create a sufficiently small kernel size to localize atoms in a cluster. Then, the localized maximum pixels are thresholded by $\text{I}_{\text{Au}}$. Since the clusters being imaged consist of atoms that are bonded and close together, individual atoms will have overlapping areas, resulting in peak pixel intensity values being higher than $\text{I}_{\text{Au}}$. Using $\text{I}_{\text{Au}}$ as a threshold therefore helps to remove the Br atoms and noise in the image stacks. After finding the atoms, the algorithm marks them with a red dot. All of the frames in the same image stack are processed with the same kernel size ($\text{KS}_{\text{Au}}$) and threshold ($\text{I}_{\text{Au}}$) value.

The algorithm for finding single atoms is summarized below:

1. Apply maximum filter (ndimage.maximum_filter) on the original image frame frame_i and create a new image frame_i’
2. Find the pixel locations where the pixel intensity is the same in frame_i and frame_i’, store the pixel locations in a list t.
3. For a pixel p which pixel location is recorded in list t, if the intensity I_p_ < I_Au_, remove the pixel in the list t.
4. Mark the pixels still in the list t on the frame_i with a red dot.

After marking the Au atoms, the number of atoms in each cluster is manually read out. Although the atom localization algorithm greatly improves the reliability of counting single atoms compared to human vision, individual frames are still prone to error. Consequently, we average the atom count values (*N*_Au_) across all frames in an image stack as clusters diffuse and rotate, capturing multiple orientations of the same structure. Statistical analysis of these values helps to account for the possibility of two atoms directly overlaying, noise, and other artifacts present in individual frames, allowing for a consistent count of the number of atoms per cluster of ≈32 (Supplementary Figure 14).

**Two-component Langmuir Model for Quantification of Ligand Binding Constants**

To extract quantitative estimates for the ratio of ligand binding constants to Au_32_ clusters we make use of the well-known Langmuir Isotherm model in which two separate adsorbates (*A* and *B*) may interact with a surface in equilibrium characterized by an equilibrium constant *K*_eq_*^A^* or *K*_eq_*^B^*, respectively:^5^

$\frac{\text{θ}_{\text{A}}}{\text{θ}_{\text{B}}}\text{=}\frac{\text{K}_{\text{eq}}^{\text{A}}{\text{[}\text{A}\text{]}}_{\text{sol}}}{\text{K}_{\text{eq}}^{\text{B}}{\text{[}\text{B}\text{]}}_{\text{sol}}}$ (Supplementary eq. 2)

where *θ_A_* and *θ_B_* denote the fraction of binding sites occupied by adsorbate *A* or adsorbate *B* and [*A*]_sol_ and [*B*]_sol_ represents their concentration in solution, respectively. We define *R* = [*A*]_sol_/[*B*]_sol_ to be the ratio of ligand concentrations in solution and impose the restriction that *θ_A_*+*θ_B_* = 1 since the Au_32_ cluster has a finite number of binding sites (20 for X^-^ halides, 12 for [AQA^+^•X^-^] bound ion pairs) that must be occupied by either adsorbate A or B:

$\frac{\text{1}}{\text{θ}_{\text{B}}}\text{-1=}\frac{\text{K}_{\text{eq}}^{\text{A}}}{\text{K}_{\text{eq}}^{\text{B}}}\text{R}$ (Supplementary eq. 3)

which shows that plots of $\frac{\text{1}}{\text{θ}_{\text{B}}}\text{-1}$ vs. *R* should yield linear functions, the slopes of which provide the ratio of equilibrium constants, *K*_eq_*^A^*/*K*_eq_*^B^*.

We determine values for *θ_B_* by integrating the intensity of MALDI mass spectrometry peaks corresponding to individual mixed-ligand clusters (Figure 4b, d) and normalizing such that all values sum to 100. We assume that the ionization potential of every mixed-ligand cluster is identical,^6-7^ which is reasonable since they have similar mass, identical charge, and their UV-vis absorption spectra are essentially overlapping (Supplementary Figure 17), indicating a similar electronic structure in each species. This allows for the normalized integrated peak intensities from MALDI to be interpreted as the relative fraction of clusters with a given ligand configuration. Plots of normalized integrated peak intensity vs. number of *B* ligands can then be fit to gaussian functions to determine a numerical value of the peak position which is then divided by 12 (in the case of [AQA^+^•X^-^] bound ion pair ligands) or 20 (in the case of X^-^ halide ligands) to find *θ_B_* (Supplementary Figure 22). Gaussian fits were constrained to have peak intensities within 10% of the largest experimental datapoint. Plots of $\frac{\text{1}}{\text{θ}_{\text{B}}}\text{-1}$ vs. *R* that were used to extract binding constant ratios are available in Supplementary Figure 23.

**Technical details of MD and DFT simulations**

The temperature was kept constant using a Langevin thermostat with a 5 ps^−1^ damping constant. In constant pressure simulations, pressure was maintained using a Langevin piston with a period of 1 ps and a decay rate of 50 fs. Nonbonded interactions between atoms within three bonds of each other, or atoms further than 12 Å from each other, were assumed to be zero. Consequently, they contributed to the calculated forces only through the long-range electrostatics described by the particle mesh Ewald method with a grid spacing of 1.0 Å. A switching function was applied to pairs of atoms between 8 and 12 Å so as to remove any long-range forces beyond 12 Å. All simulations employed periodic boundary conditions and were propagated with a 2 fs timestep. Visual molecular dynamics (VMD) was used to solvate Au_32_ systems with TIP3P water within cubic boxes with 100 Å side lengths.^8-9^ First, the system was minimized (100 000 steps) using conjugate gradient minimization. Then, 1 ns NPT (1 atm, 300 K) equilibration was performed. Finally, we ran the production runs for 1.6 ns in NVT ensemble.

From these simulations, the Au_32_Cl_8_[C_16_TA^+^•Cl^-^]_12_ near equilibrium was used to perform the calculations to determine the optical spectrum of Au_32_Cl_8_[C_16_TA^+^•Cl^-^]_12_. DFT and TD-DFT calculations were performed for the Au_32_Cl_8_[NH_4_^+^•Cl^-^]_12_ using the Amsterdam Density Functional (ADF) 2020 package^10^ at the BP86^11-12^/TZP^13^ level of theory. The frozen core approximation was applied to the core electrons of all elements and the ZORA scalar relativistic Hamiltonian^14-16^ was applied for the gold atoms. For geometry optimization, the gold atoms were constrained in all directions and all other atoms were allowed to move freely until the energy and gradient convergences were met, 1 x 10^-4^ and 1x 10^-3^ respectively. The optical spectrum was obtained at the same level of theory.

Supplementary Figure 17 shows the spectra collected from simulations and experiment of Au_32_X_8_[NH_4_^+^•X^-^]_12_ (X = Cl, Br). The simulated spectrum for Au_32_Cl_8_[C_16_TA^+^•Cl^-^]_12_ contains peaks at 831 nm, 699 nm, 615 nm, and 386 nm with a shoulder at 436 nm. By comparison, the experimentally collected UV-Vis spectra has peaks at 806 nm, 639 nm, and 391 nm, with a shoulder at 513 nm. It should be noted that the two simulated peaks observed at 699 and 615 nm could contribute to the broad peak at 639 nm in the spectrum collected from experiment. The less intense peaks observed at 831, 699, and 615 nm contain an electron-hole pair whose densities are delocalized throughout the gold core, with minimal electron density on the Cl-atoms. The most intense peak along with its shoulder, e.g. 386 nm and 464 nm, originate from localized atomic d-orbitals and transition to states with density delocalization on the gold core with contributions from the five lowest unoccupied states (Supplementary Table 2). Thus, the similarity of absorption spectra from Au_32_ clusters with different halide and bound ion pair ligands is expected.

**Supplementary Table 2.** Contributions of orbital transitions to main observed peaks from TD-DFT simulations. Only transitions with values above 10% are listed.

| **λ (nm)** | **Orbital Transition** | | **Percentage Intensity at λ** |
| --- | --- | --- | --- |
|  | **From** | **To** |  |
| 831 | HOMO -5  (423) | LUMO  (429) | 77.5% |
| 831 | HOMO -4  (424) | LUMO  (429) | 12.8% |
| 699 | HOMO -3  (425) | LUMO +4  (433) | 40.7% |
| 699 | HOMO -11  (419) | LUMO  (429) | 20.5% |
| 699 | HOMO -5  (423) | LUMO +1  (430) | 13.0 |
| 436 | localized d-orbitals  (376) | LUMO +2  (431) | 24.4 |
| 436 | localized d-orbitals (377) | LUMO +3  (432) | 14.1 |
| 386 | localized d-orbitals  (344) | LUMO  (429) | 37.8 |
| 386 | HOMO -6  (422) | LUMO +7  (436) | 19.4 |

**Discussion of MALDI-ToF-MS Characterization of Au_32_ Nanoclusters**

Matrix-Assisted Laser Desorption/Ionization (MALDI) is a well-studied soft ionization technique that has been successfully applied to the investigation of gold clusters^17^. However, reasonable concerns could be raised regarding the possibility that the intense laser excitation might ablate a set of precursors dried on the sample plate and generate the observed gold clusters in the gas phase. To probe the solution-phase existence and stability of the clusters observed in MALDI, two control experiments were conducted. Both rely on the hypothesis that if clusters were forming in the gas phase during laser excitation, two or more separate samples with identical concentrations of all components should produce identical MALDI spectra. Therefore, if a single analyte solution can be shown to produce mass spectra that shifts over time (e.g., by observing the kinetics of a ligand exchange reaction), this would strongly suggest that a cluster-based reaction is occurring in solution and being kinetically resolved. Alternatively, if two different analyte solutions are sequentially deposited on a MALDI plate, the order in which they are deposited should not matter if the components are all excited into the gas phase where they mix and form clusters. However, if the order of sequential deposition produces different mass spectra, it would strongly suggest that a ligand exchange reaction was occurring during the process of drying the solution on the sample plate which necessitates the pre-existence of clusters before laser excitation. Both experiments confirm the solution-phase existence of Au_32_ clusters and are described in more detail below.

In the first set of experiments, Au_32_Br_8_[C_16_TA^+^•Br^-^]_12_ clusters were added to a solution of C_14_TAB to initiate a ligand exchange reaction (Supplementary Figure 19a). A series of mixed-ligand Au_32_Br_8_[C_14_TA^+^•Br^-^]_12-_*_x_*[C_14_TA^+^•Br^-^]*_x_* clusters formed immediately upon the mixing (Supplementary Figure 19b). However, as the reaction proceeds from 0 to 30 m, the distribution of mixed-ligand cluster peaks shifts towards Au_32_Br_8_[C_14_TA^+^•Br^-^]_12_ (Supplementary Figure 19 b-e). Note that the peak associated with Au_32_Br_8_[C_14_TA^+^•Br^-^]_11_[C_14_TA^+^•Br^-^]_1_ becomes more significant over time. Since each sample in Supplementary Figure 19 contains an identical concentration of reagents, the shifting mass spectra is most consistent with the presence of a solution-phase Au_32_ cluster undergoing a ligand exchange reaction.

In the second set of experiments, a Au_32_Br_8_[C_14_TA^+^•Br^-^]_12_ cluster solution (synthesized with 25 mM C_14_TAB) and a 25 mM C_16_TAB surfactant solution were dried on the same spot on the MALDI target plate and compared to a spot where a Au_32_Br_8_[C_16_TA^+^•Br^-^]_12_ cluster solution (synthesized with 25 mM C_16_TAB) was dried with a 25 mM C_14_TAB surfactant solution. Once dried, these two spots consist of the same number of Au atoms, halides, surfactant molecules, and reducing agent components. Nonetheless, the two mass spectra show different distributions of hybrid clusters (Supplementary Figure 9). For the mixture of Au_32_Br_8_[C_14_TA^+^•Br^‑^]_12_ cluster solution and C_16_TAB surfactant solution (Supplementary Figure 9b), the dominant peak is Au_32_Br_8_[C_14_TA^+^•Br^‑^]_12_. However, for the mixture of Au_32_Br_8_[C_16_TA^+^•Br^-^]_12_ cluster solution and C_14_TAB surfactant solution (Supplementary Figure 9c), the dominant peak is Au_32_Br_8_[C_16_TA^+^•Br^‑^]_12_. These differences suggest that a ligand exchange reaction is occurring once the surfactant solution is added to the dried cluster solution. The presence of solution-phase Au_32_ clusters with different alkyquaternaryammonium ligands in the initial sample is most consistent with these data.

We also note here that both Au_32_Br_8_[C_14_TA^+^•Br^-^]_12_ and Au_32_Br_8_[C_16_TA^+^•Br^-^]_12_ clusters show identical mass spectra when prepared using two different MALDI matrix molecules, DCTB and TA9 (Supplementary Figure 8). This indicates that the matrix does not form an adduct with the cluster and thus the assignment of the measured mass, after subtracting the mass of the ligands, entirely to Au atoms is justified.

We conclude from these experiments that the identified Au_32_ clusters are indeed generated in solution as a result of the traditional seed synthesis and MALDI-ToF-MS characterization of their mass and ligand exchange is appropriate.

SUPPLEMENTARY FIGURES


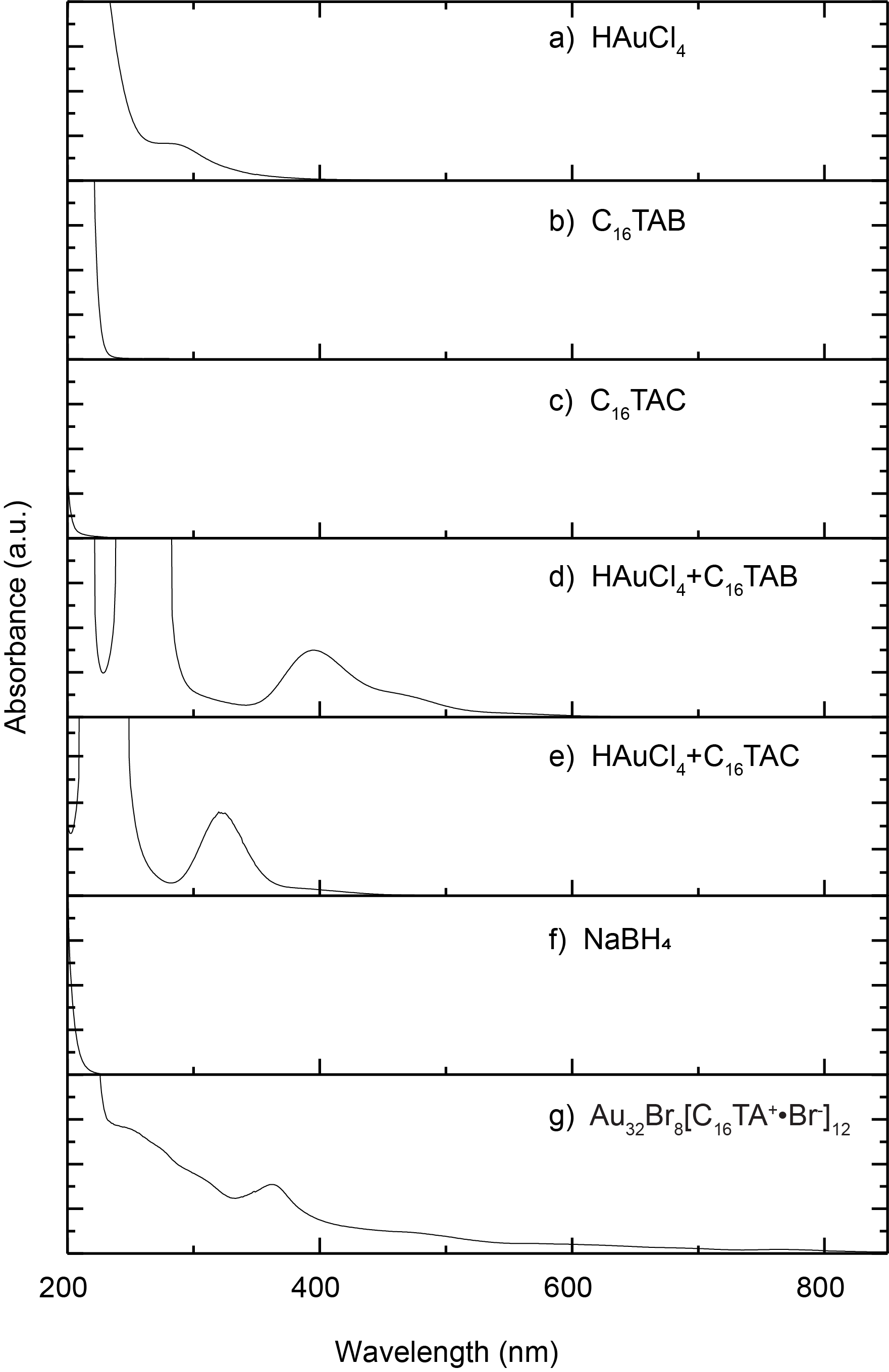


**f** NaBH_4_

**g** Au_32_Br_8_[C_16_TA^+^•Br^-^]_12_

**d** HAuCl_4_ + C_16_TAB

**e** HAuCl_4_ + C_16_TAC

**c** C_16_TAC

**b** C_16_TAB

**a** HAuCl_4_

**Supplementary Figure 1.** UV-Vis-NIR absorbance of **a** HAuCl_4_, **b** C_16_TAB, **c** C_16_TAC, **d** HAuCl_4_+C_16_TAB, **e** HAuCl_4_+C_16_TAC, **f** NaBH_4_, and **g** Au_32_Br_8_[C_16_TA^+^•Br^-^]_12_. None of the reagent traces has peaks that coincide with the Au_32_Br_8_[C_16_TA^+^•Br^-^]_12_ sample **g**, i.e., 365 nm, 480 nm, and 580 nm.


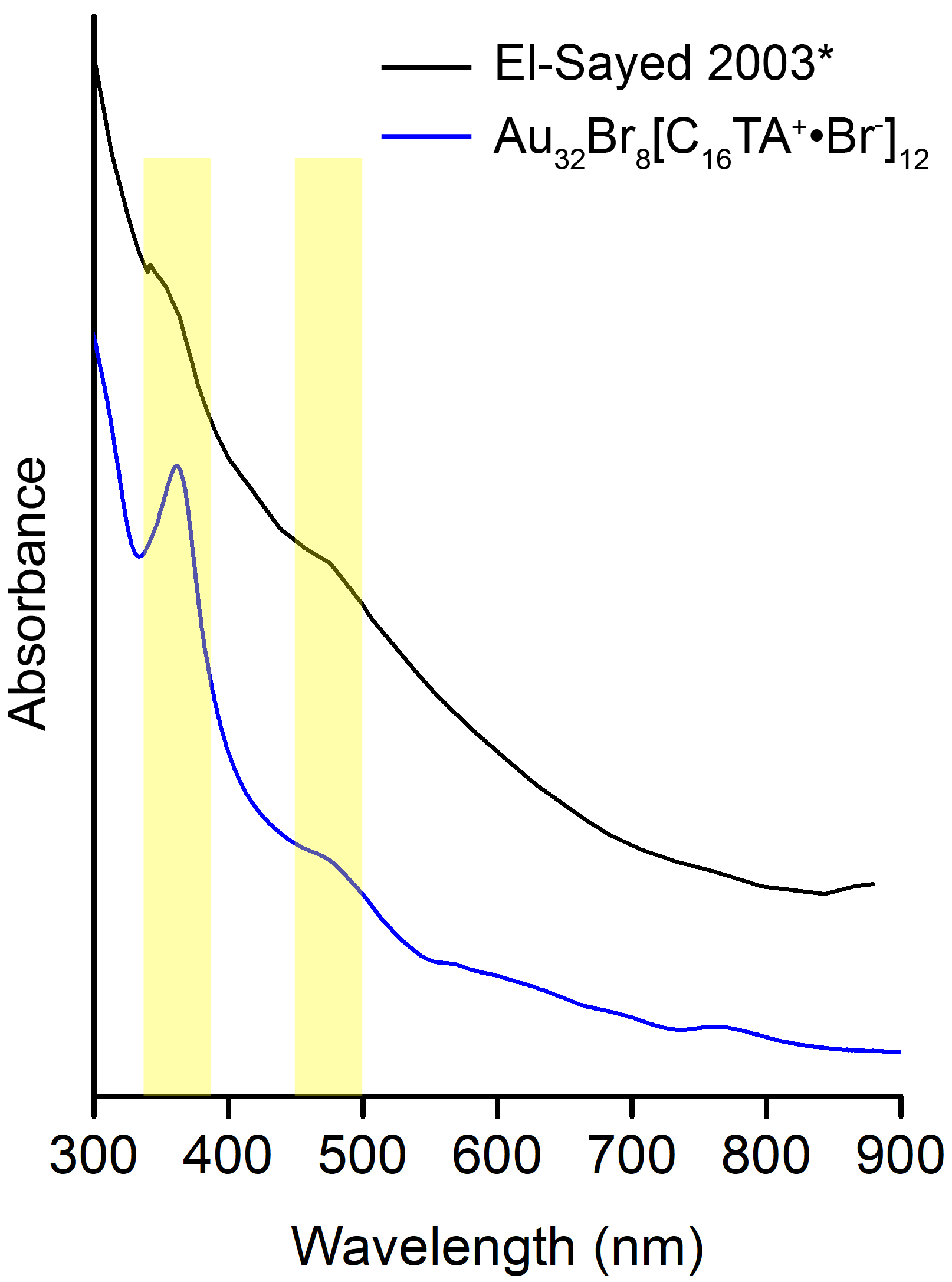


**Supplementary Figure 2.** Comparison of the absorption spectra of Au_32_ clusters reported in this work (blue) to first known example of aqueous seeds synthesized in C_16_TAB solution (black, *El-Sayed and coworkers, *Chem. Mater.* **2003,** *15*, 1957-1962). Data from Figure 1 of El-Sayed 2003 extracted using WebPlotDigitizer v4.4. Yellow bars are 50 nm in width and centered on the most pronounced optical transitions that appear in both samples.


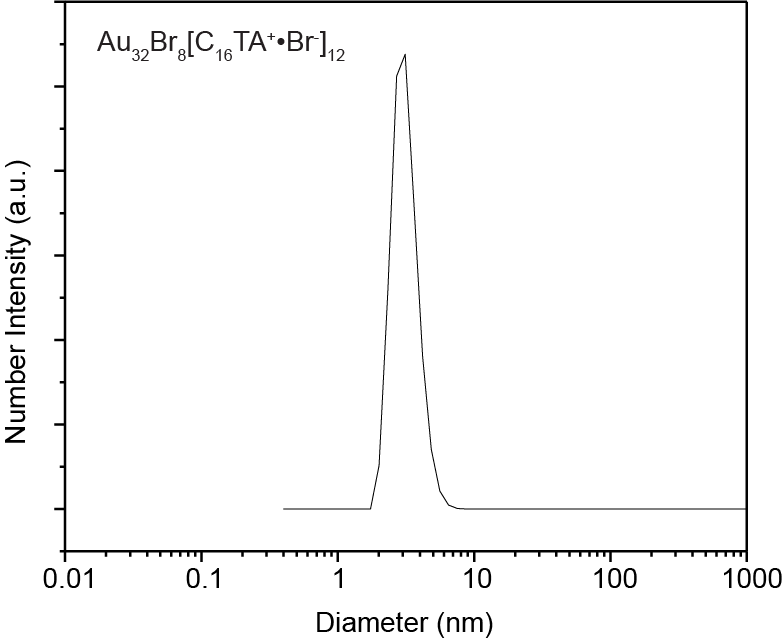


**Supplementary Figure 3.** Dynamic light scattering (DLS) of Au_32_Br_8_[C_16_TA^+^•Br^-^]_12_ solutions after being spun down at 4 ºC. As-prepared Au_32_Br_8_[C_16_TA^+^•Br^-^]_12_ samples have roughly 50 mM C_16_TAB. Cooling down to 4 ºC and centrifuging at 20k RCF for 30 min precipitates over 49 mM C_16_TAB. The concentration of the remaining C_16_TAB is therefore below the critical micelle concentration of C_16_TAB,^18^ so no micelles exist in the supernatant solution. The existence of uniform objects in the DLS data suggests the presence of cluster species.


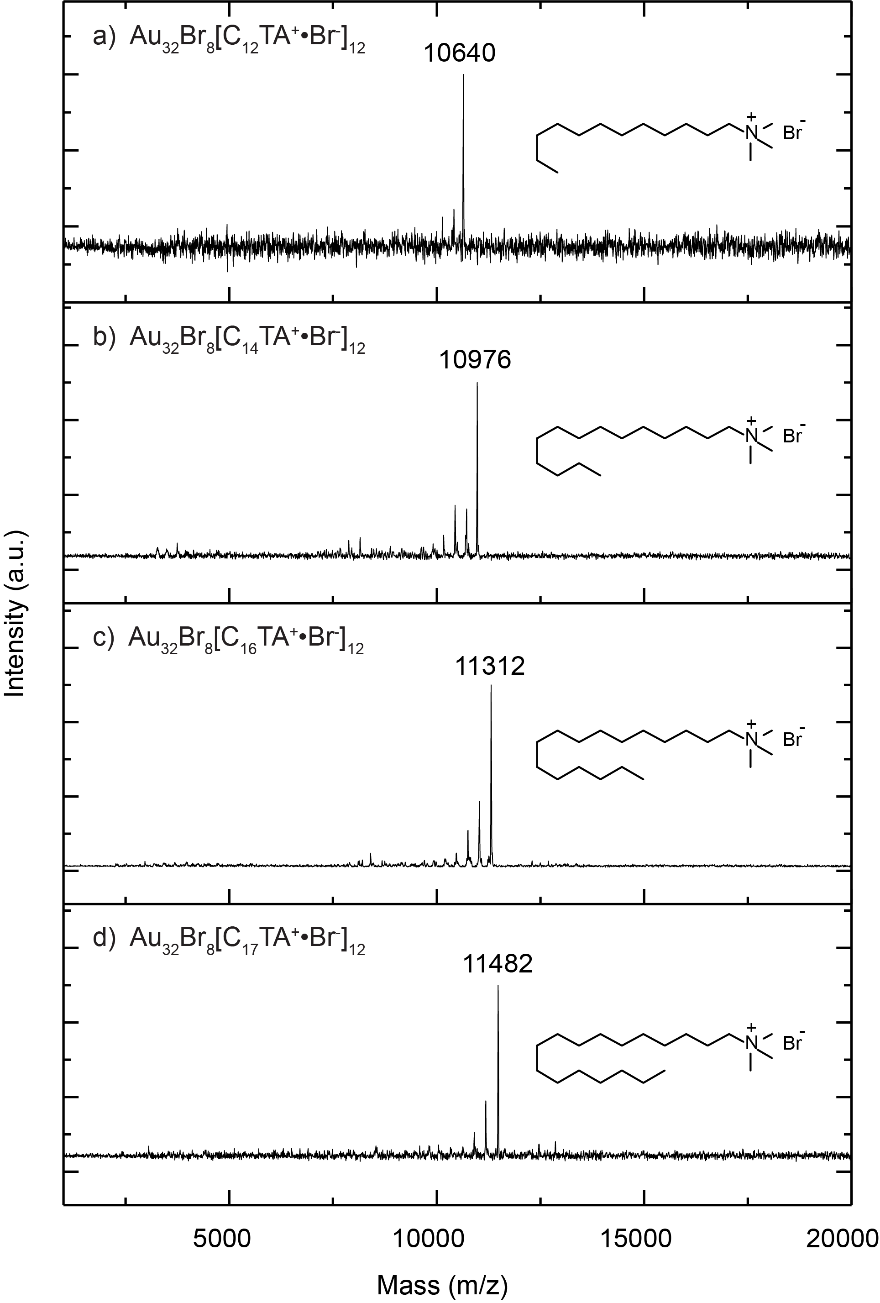


**d** Au_32_Br_8_[C_17_TA^+^•Br^-^]_12_

**c** Au_32_Br_8_[C_16_TA^+^•Br^-^]_12_

**b** Au_32_Br_8_[C_14_TA^+^•Br^-^]_12_

**a** Au_32_Br_8_[C_12_TA^+^•Br^-^]_12_

(*m*/*z*)

**Supplementary Figure 4.** Mass spectra of **a** Au_32_Br_8_[C_12_TA^+^•Br^-^]_12_, **b** Au_32_Br_8_[C_14_TA^+^•Br^-^]_12_, **c** Au_32_Br_8_[C_16_TA^+^•Br^‑^]_12_, and **d** Au_32_Br_8_[C_17_TA^+^•Br^-^]_12_ over a wide range of *m/z* values. The absence of additional peaks indicates that Au_32_ is the dominant cluster product of the seed synthesis.


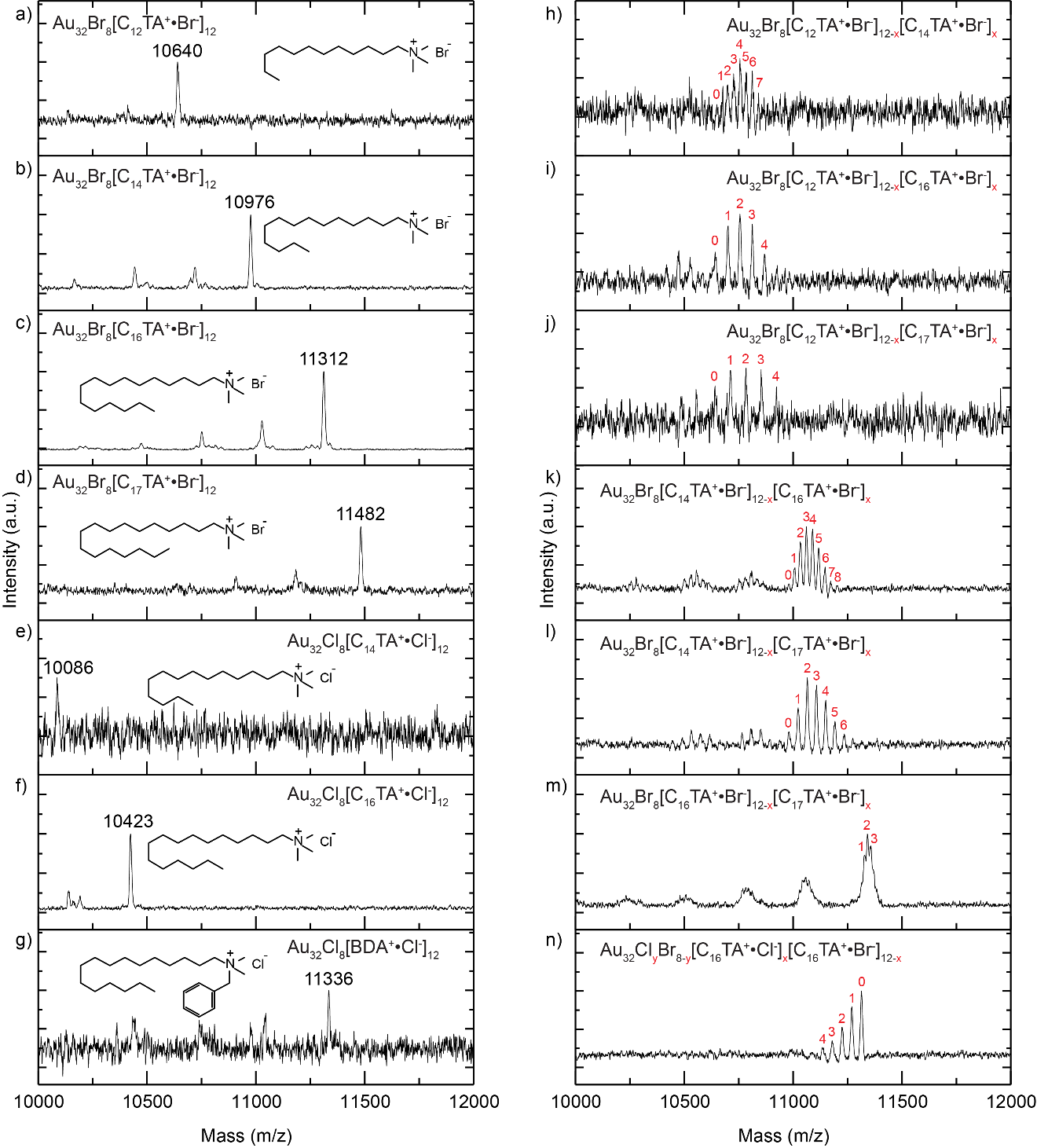


**n**

**m**

**l**

**k**

**j**

**i**

**h**

**g**

**f**

**e**

**d**

**c**

**b**

**a**

(*m*/*z*)

(*m*/*z*)

**Supplementary Figure 5.** Library of Au_32_ nanoclusters synthesized with pure or mixed ligand shells. Pure ligands (top to bottom): **a** Au_32_Br_8_[C_12_TA^+^•Br^-^]_12_, **b** Au_32_Br_8_[C_14_TA^+^•Br^-^]_12_, **c** Au_32_Br_8_[C_16_TA^+^•Br^-^]_12_, **d** Au_32_Br_8_[C_17_TA^+^•Br^‑^]_12_, **e** Au_32_Cl_8_[C_14_TA^+^•Cl^-^]_12_, **f** Au_32_Cl_8_[C_16_TA^+^•Cl^‑^]_12_, and **g** Au_32_Cl_8_[BDA^+^•Cl^-^]_12_. Mixed ligands (top to bottom): **h** Au_32_Br_8_[C_12_TA^+^•Br^-^]*_x_*[C_14_TA^+^•Br^-^]_12-_*_x_*, **i** Au_32_Br_8_[C_12_TA^+^•Br^-^]*_x_*[C_16_TA^+^•Br^-^]_12-_*_x_*, **j** Au_32_Br_8_[C_12_TA^+^•Br‑]*_x_*[C_17_TA^+^•Br^‑^]_12‑_*_x_*, **k** Au_32_Br_8_[C_14_TA^+^•Br^-^]*_x_*[C_16_TA^+^•Br^-^]_12-_*_x_*, **l** Au_32_Br_8_[C_14_TA^+^•Br‑]*_x_*[C_17_TA^+^•Br^‑^]_12-_*_x_*, **m** Au_32_Br_8_[C_16_TA^+^•Br‑]*_x_*[C_17_TA^+^•Br^-^]_12-_*_x_*, and **n** Au_32_Cl*_y_*Br_8‑_*_y_*[C_16_TA^+^•Cl^‑^]*_x_*[C_16_TA^+^•Br^‑^]_12-_*_x_*. *x* (or *x*+*y*, if *y* is present) in the formula is labeled in red and placed over the peaks of hybrid clusters.


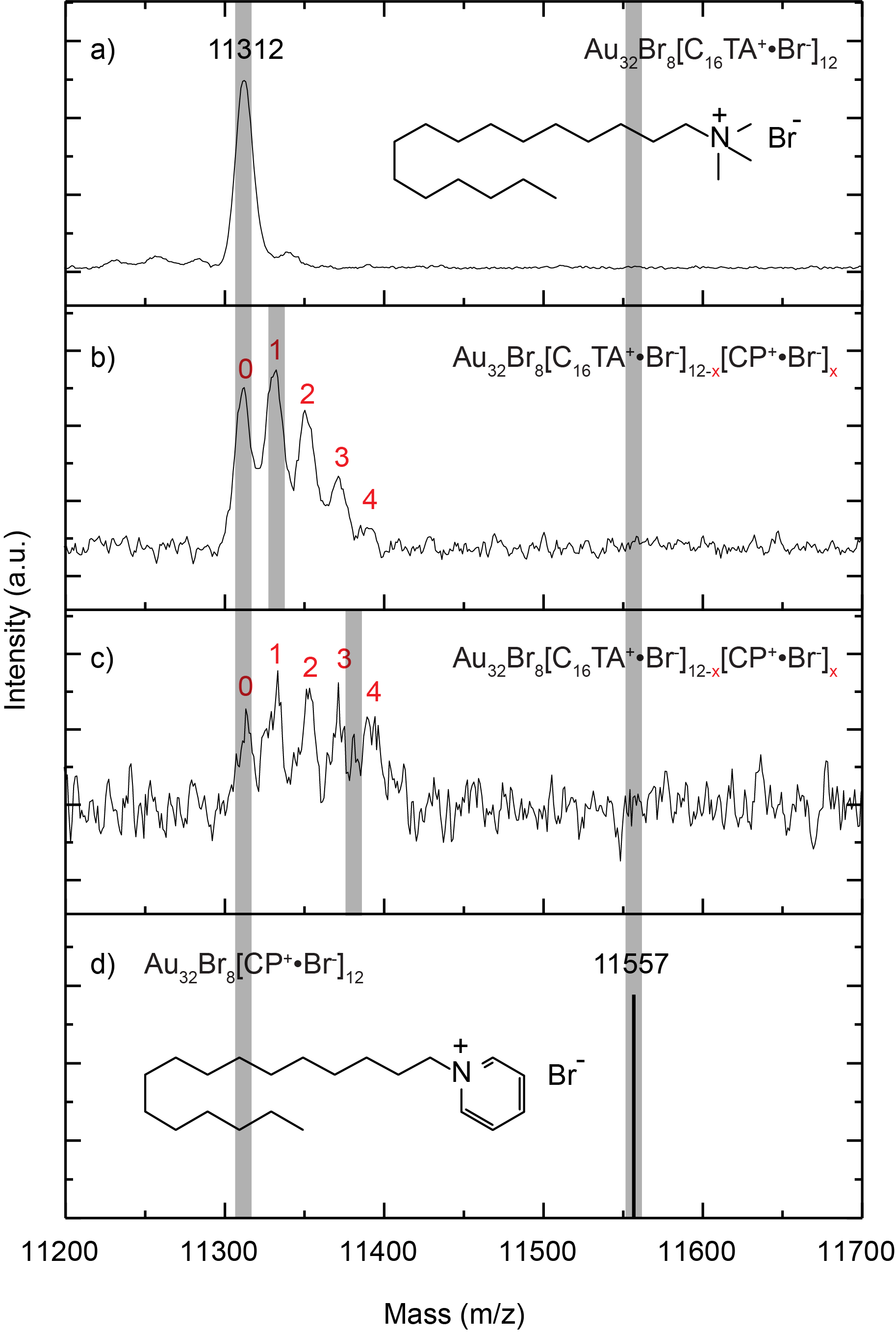


**d**

**c**

**b**

**a**

(*m*/*z*)

**Supplementary Figure 6.** Ligand exchange of C_16_TAB and CPB at various ratios. **a** Au_32_Br_8_[C_16_TA^+^•Br^-^]_12_, **b** clusters synthesized with 45 mM C_16_TAB with CPB added to 5 mM, **c** clusters synthesized with 35 mM C_16_TAB with CPB added to 15 mM, and **d** predicted mass of Au_32_Br_8_[CP^+^•Br^-^]_12_. The nominal ratio of ligands in the solution is indicated with the short bars in **b** and **c**. The peak distribution is clearly biased toward C_16_TAB. *x* in the formula is labeled in red and placed over the peaks of hybrid clusters.


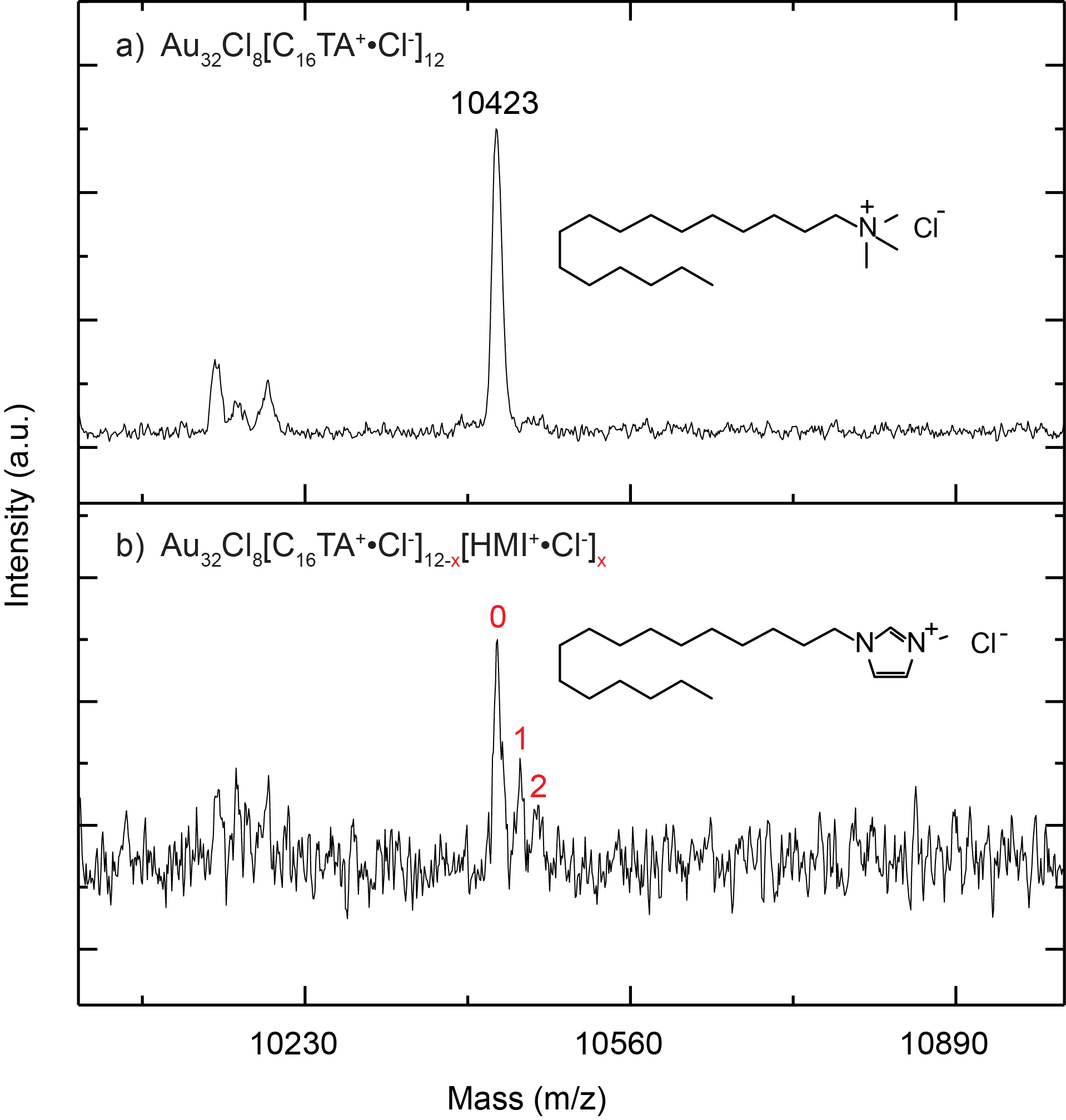


**b**

**a**

(*m*/*z*)

**Supplementary Figure 7.** Ligand exchange of C_16_TAC with 1-hexadecyl-3-methylimidazolium (HMI^+^). **a** Au_32_Cl_8_[C_16_TA^+^•Cl^-^]_12_, **b** Au_32_Cl_8_[C_16_TA ^+^•Cl^-^]_12‑_*_x_*[HMI^+^•Cl^-^]*_x_* cluster synthesized with 45 mM C_16_TAC with HMIC added to 5 mM. *x* in the formula is labeled in red and placed over the peaks of mixed ligand clusters.


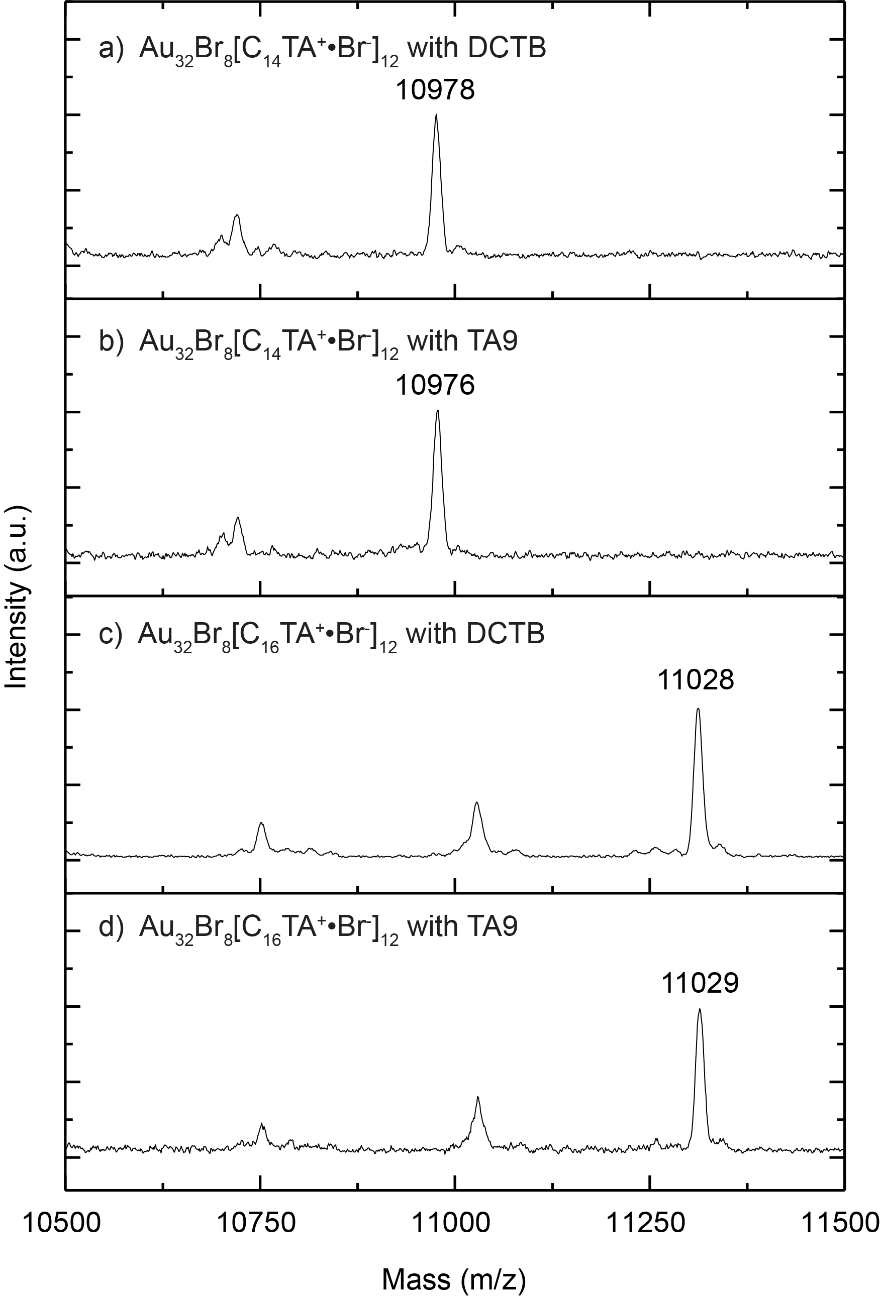


**a**

**d**

**c**

**b**

(*m*/*z*)

**Supplementary Figure 8.** Au_32_Br_8_[C_14_TA^+^•Br^-^]_12_ and Au_32_Br_8_[C_16_TA^+^•Br^-^]_12_ clusters detected in MALDI-ToF-MS with DCTB or TA9 as matrices. Au_32_Br_8_[C_14_TA^+^•Br^-^]_12_ with **a** DCTB and **b** TA9, where the major peaks overlap. Au_32_Br_8_[C_16_TA^+^•Br^-^]_12_ with **c** DCTB and **d** TA9, where the major peaks also overlap. These two pairs indicate the absence of matrix-analyte adducts.


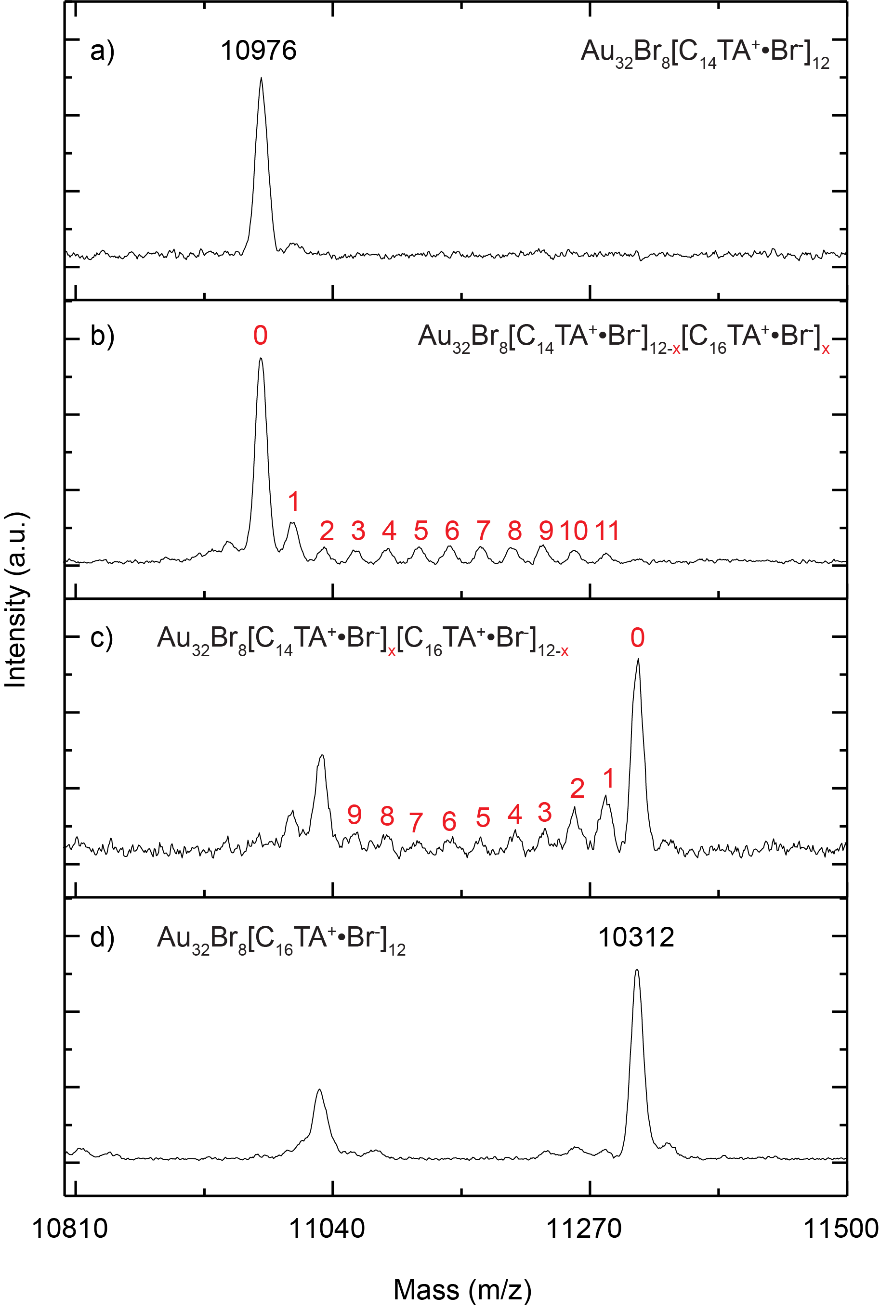


**b**

**d**

**c**

**a**

(*m*/*z*)

**Supplementary Figure 9.** On-plate ligand exchange: C_14_TAB vs C_16_TAB. **a** Au_32_Br_8_[C_14_TA^+^•Br^-^]_12_, **b** Au_32_Br_8_[C_14_TA^+^•Br^-^]_12_ synthesized with 25 mM C_14_TAB and then exchanged with 25 mM C_16_TAB on MALDI target plate, i.e., Au_32_Br_8_[C_14_TA^+^•Br^-^]_12-_*_x_*[C_16_TA^+^•Br^-^]*_x_*, **c** Au_32_Br_8_[C_16_TA^+^•Br^-^]_12_ synthesized with 25 mM C_16_TAB and then exchanged with 25 mM C_14_TAB on MALDI target plate, i.e., Au_32_Br_8_[C_14_TA^+^•Br^-^]*_x_*[C_16_TA^+^•Br^-^]_12-_*_x_*, and **d** Au_32_Br_8_[C_16_TA^+^•Br^-^]_12_. *x* in the formula is labeled in red and placed over the peaks of hybrid clusters.


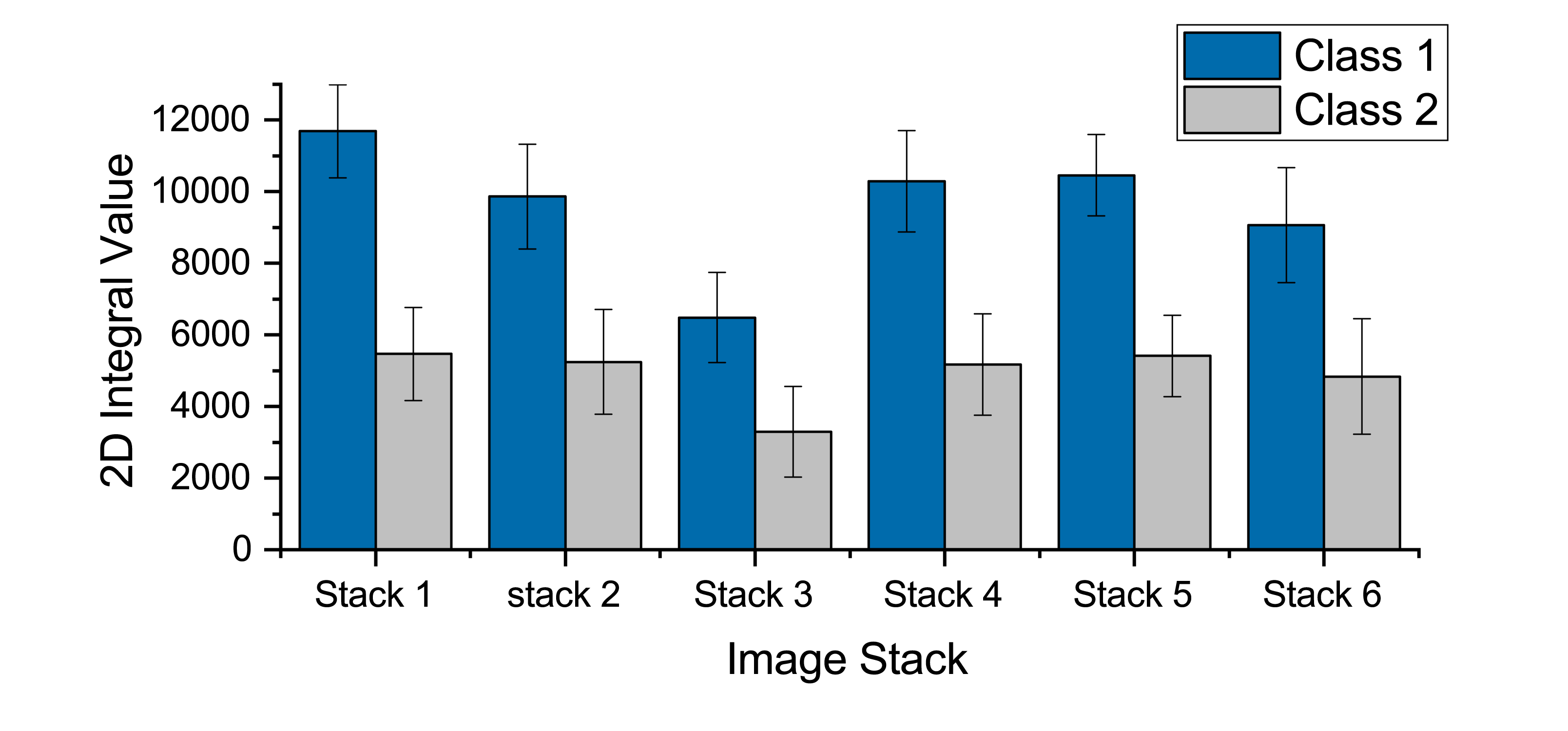


**Supplementary Figure 10.** Classification of single atom pixel intensities from ADF-STEM images shows two discrete groups (class 1 and class 2) by a k-means clustering algorithm. Error bars represent +/- standard deviation. We hypothesize these correspond to single Au or Br atoms, respectively, and use the values extracted from the former to calibrate our atom counting algorithm.


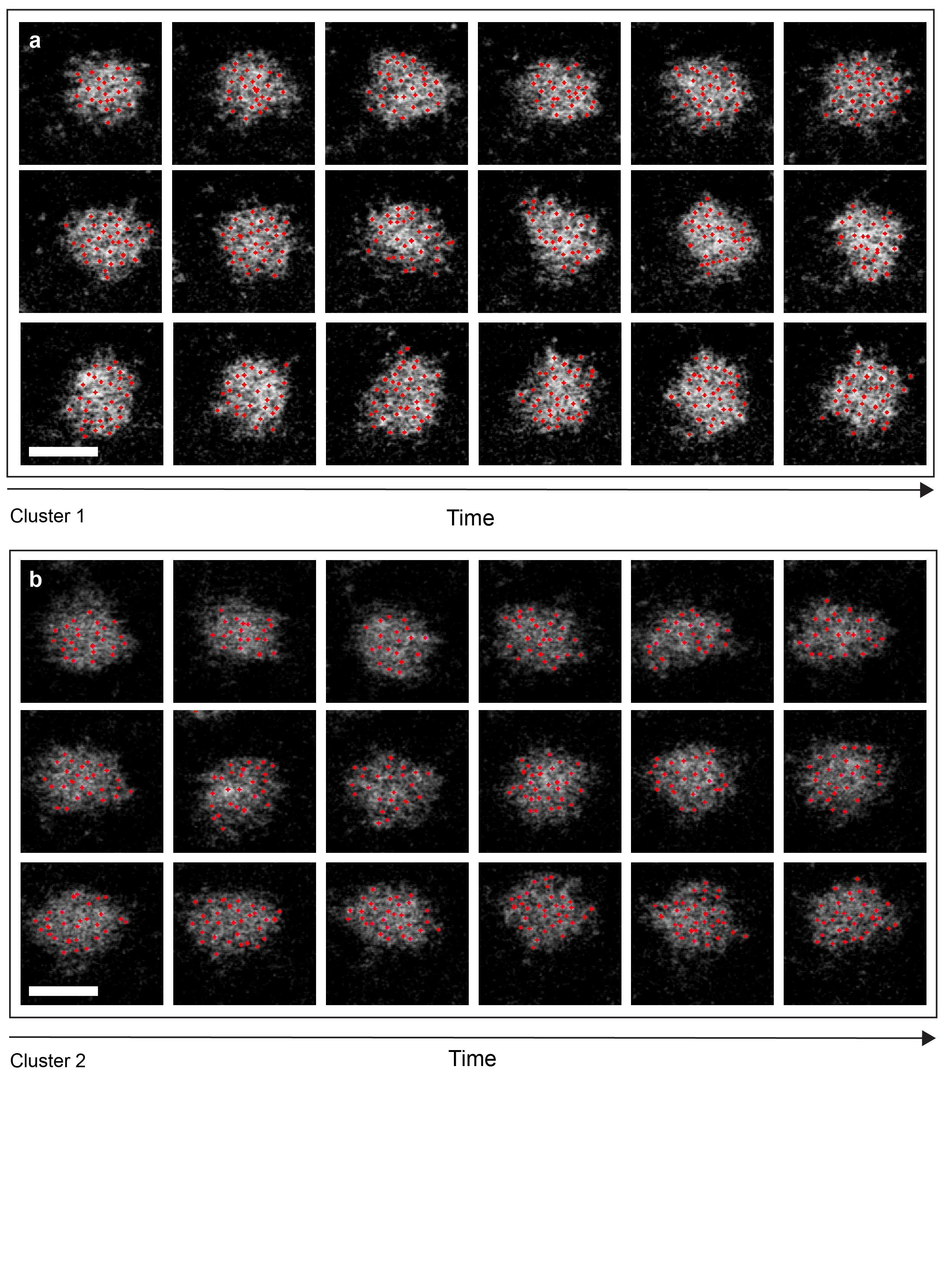


**Supplementary Figure 11.** Image stacks for Cluster 1 (top) and Cluster 2 (bottom). Scale bars: 1 nm.

**Supplementary Figure 12.** Image stacks for Cluster 3 (top) and Cluster 4 (bottom). Scale bars: 1 nm.


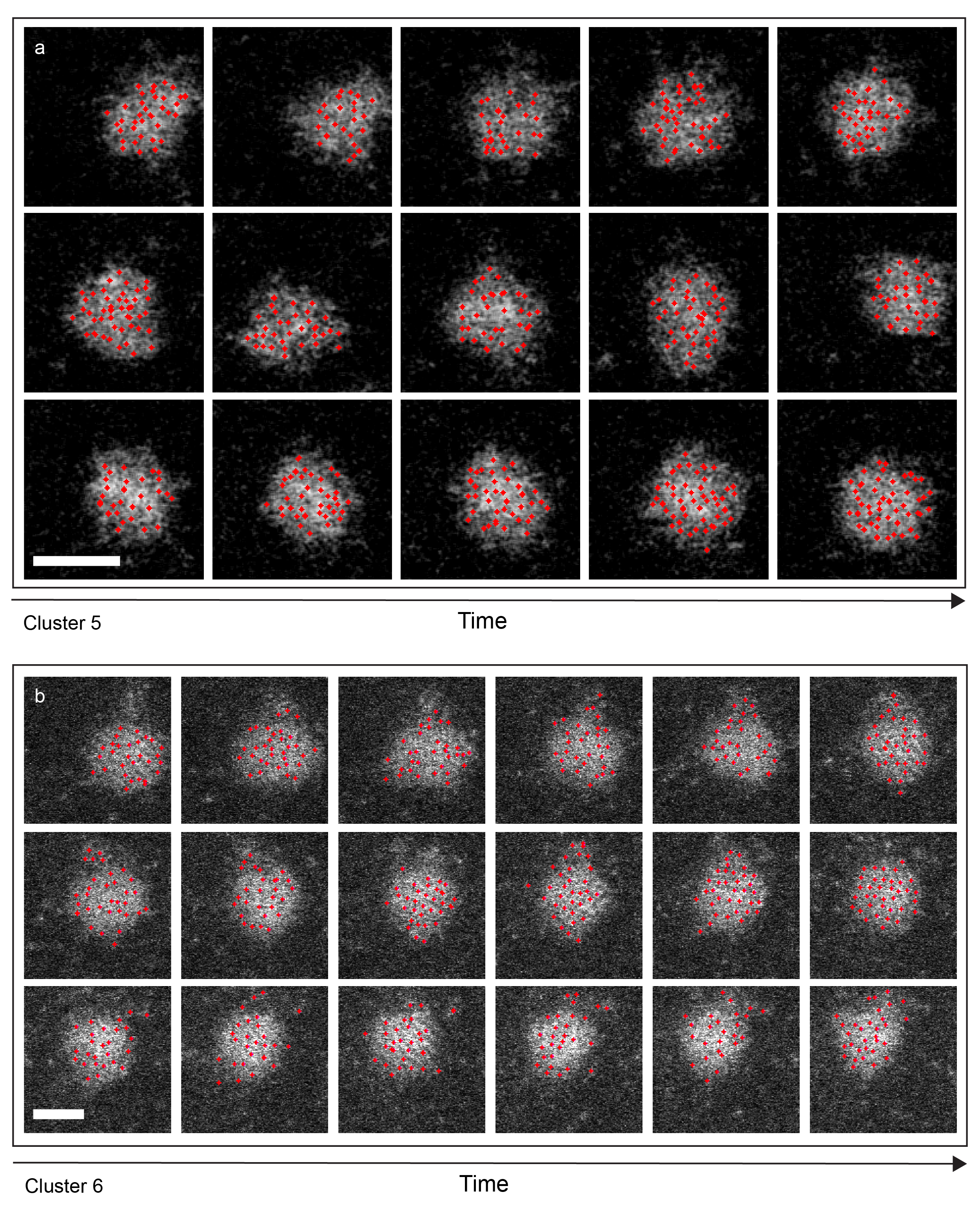


**a**

**b**

**Supplementary Figure 13.** Image stacks for Cluster 5 (top) and Cluster 6 (bottom). Scale bars: 1 nm.


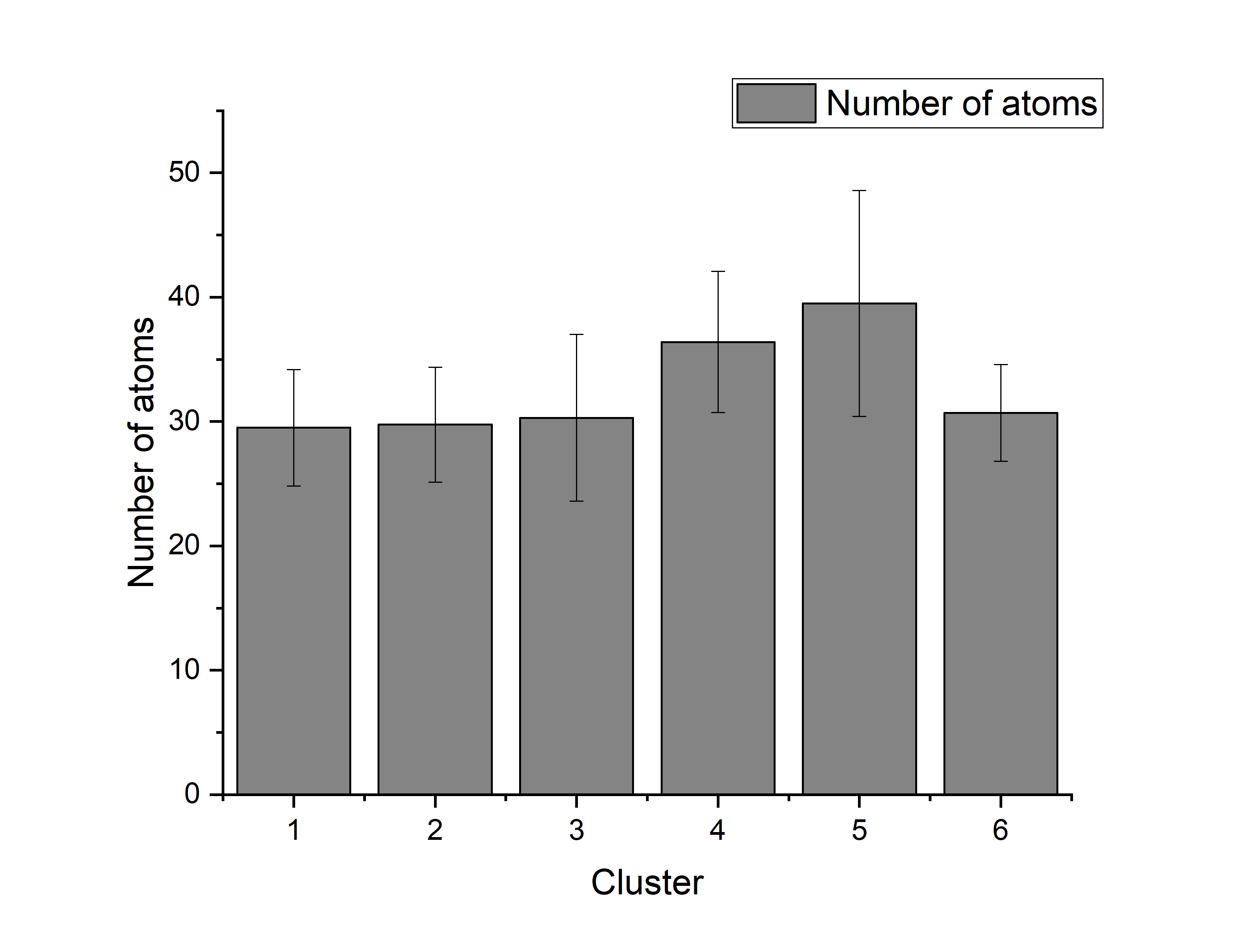


**Supplementary Figure 14.** Average number of atoms in each cluster determined by the image processing algorithm described above for the clusters presented in Supplementary Figures 11, 12, and 13. Error bars represent +/- standard deviation.

**c**


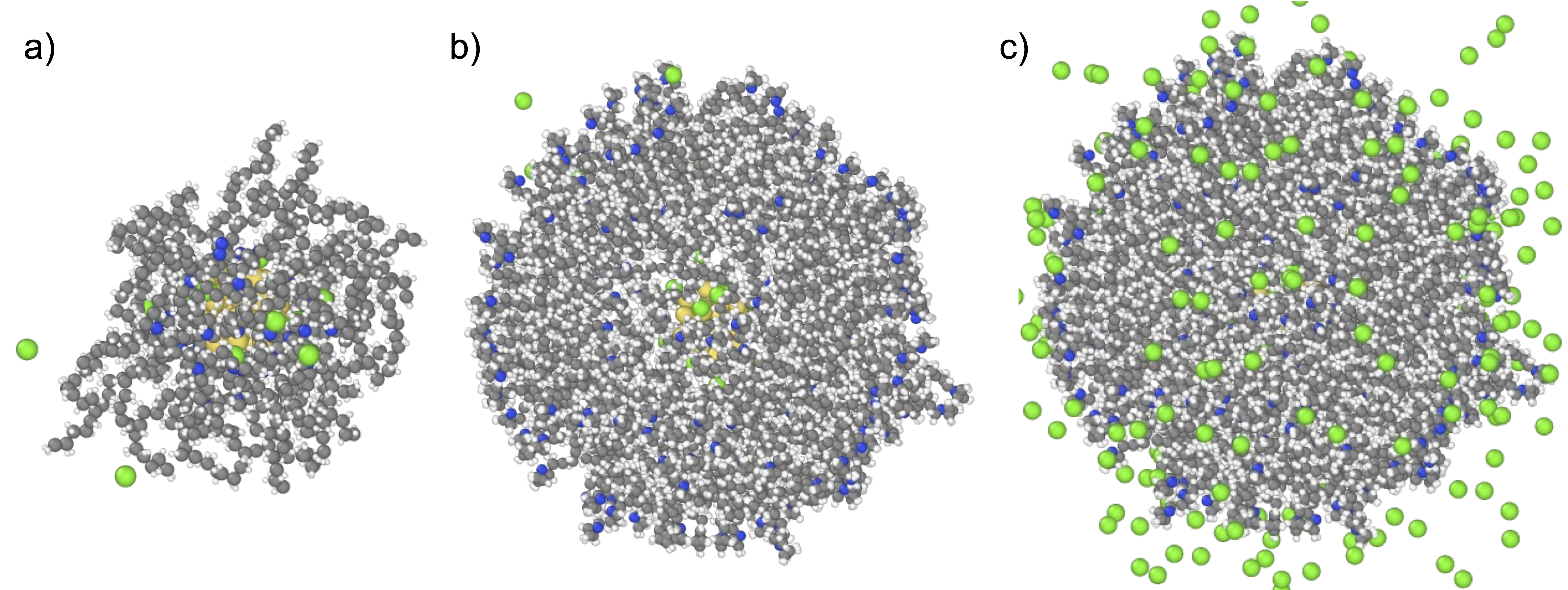


**a**

**b**

**Supplementary Figure 15.** Snapshots of Au_32_ cluster taken during the MD simulations. **a** With CTABs, 12 bound inner :26 unbound inner. **b** With 200 outer CTABs only. **c** With CTABs, 12 bound inner :26 unbound inner :200 outer. [Au:yellow, Br:green, C:grey, N:blue, H:white]


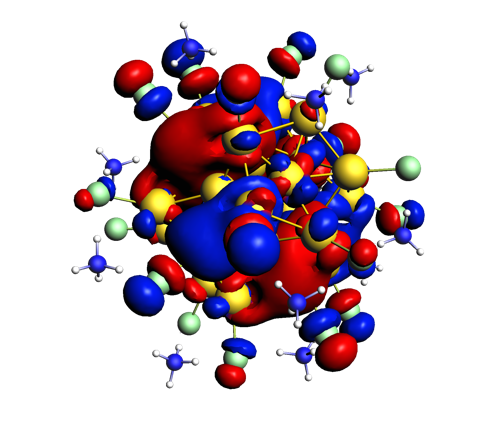

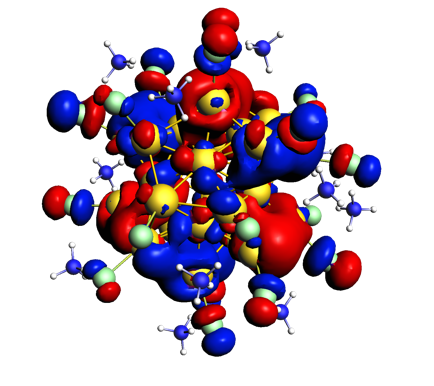


LUMO HOMO

**Supplementary Figure 16.** Main Contributing Orbitals at 386 nm for the Au_32_Cl_8_[C_16_TA^+^•Cl^-^]_12_ system.


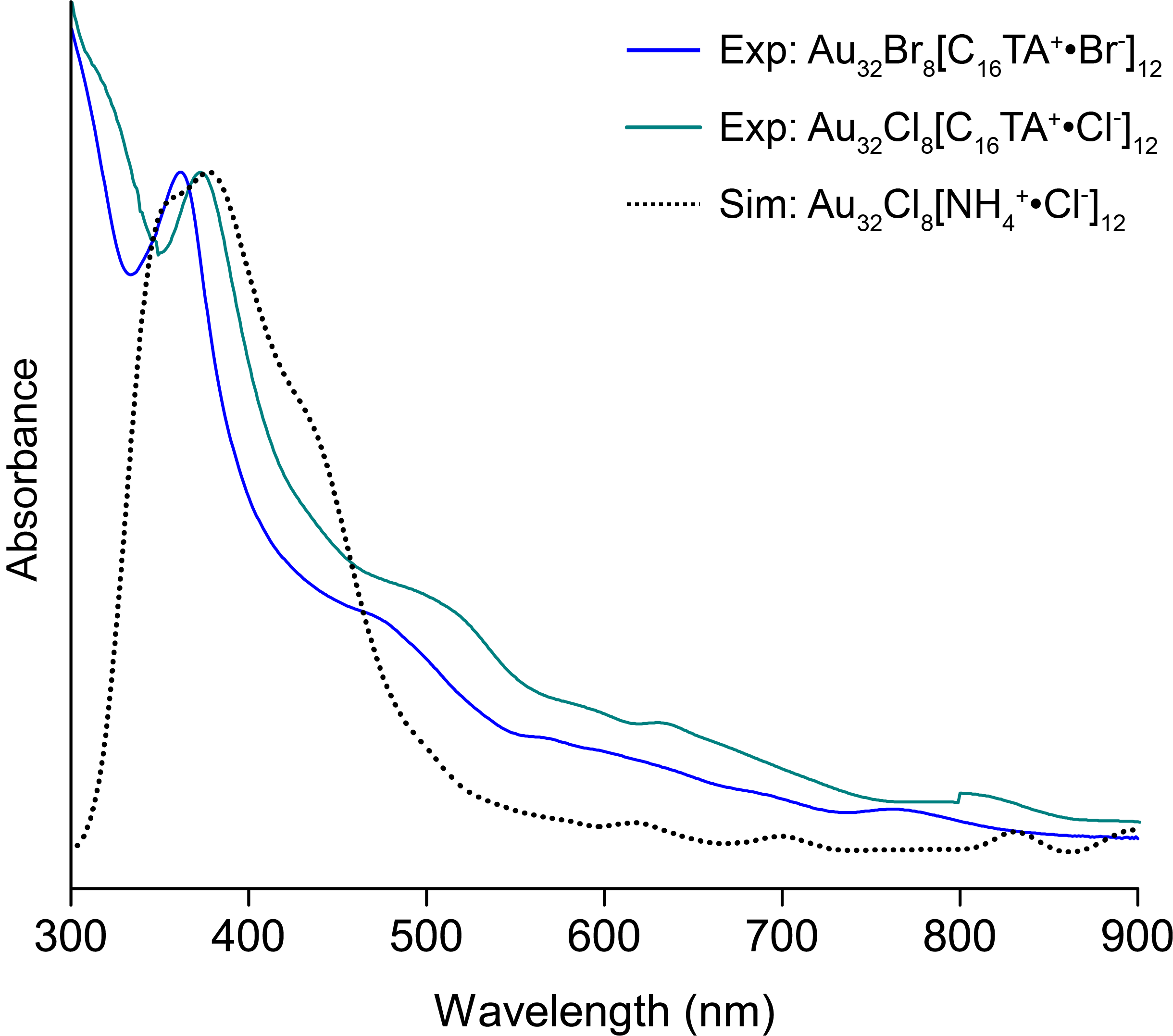


**Supplementary Figure 17.** Comparison of experimental absorption spectra of Au_32_Br_8_[C_16_TA^+^•Br^-^]_12_ (blue) and Au_32_Cl_8_[C_16_TA^+^•Cl^-^]_12_ (green) to simulated TD-DFT absorption spectra for Au_32_Cl_8_[NH_4_^+^•Cl^-^]_12_ (black dotted). The similar energy of numerous optical modes across different ligand motifs suggests a structurally similar Au_32_ core in each sample.


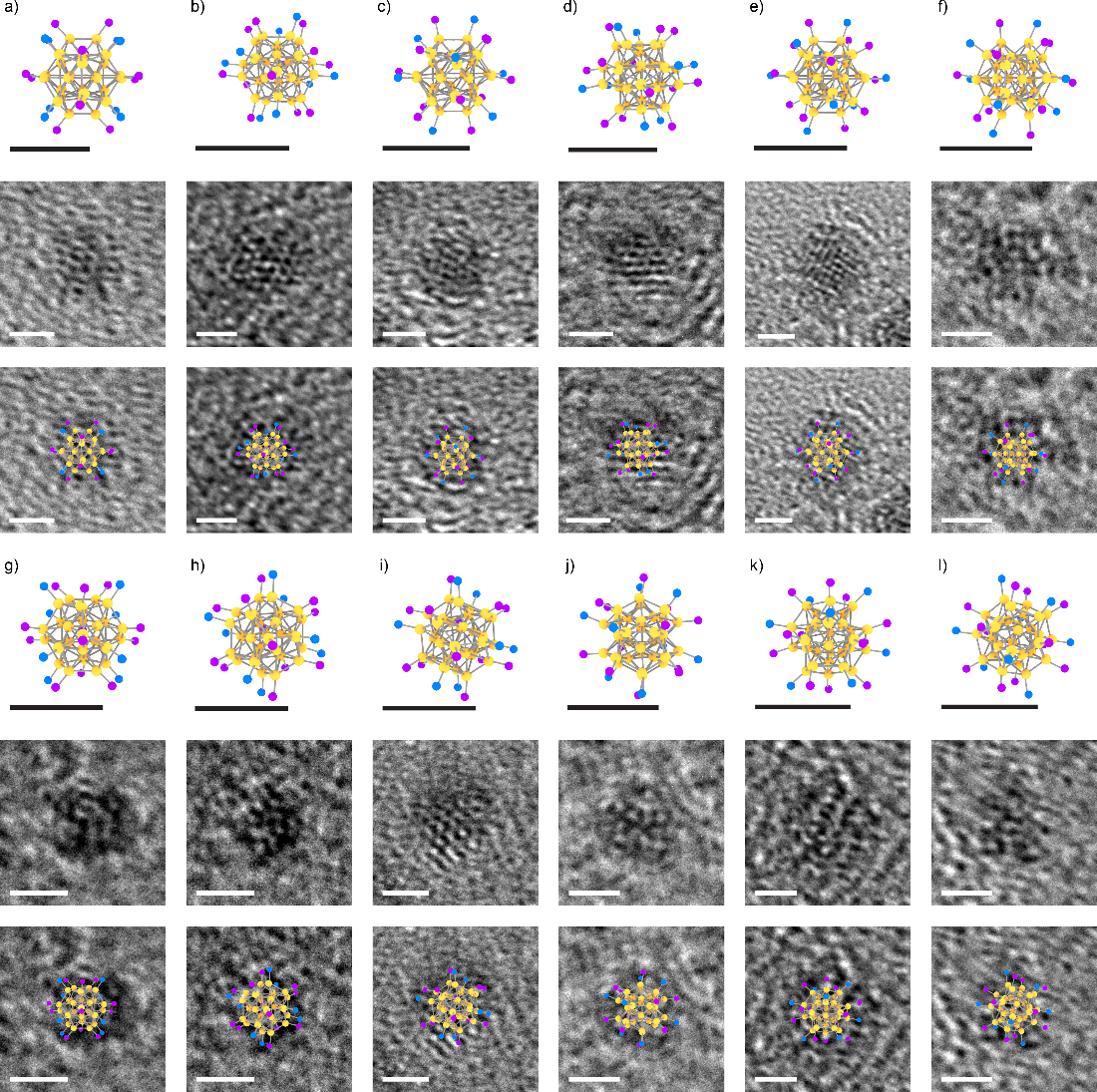


**l**

**f**

**i**

**h**

**g**

**j**

**k**

**e**

**d**

**a**

**b**

**c**

**Supplementary Figure 18.** HRTEM images of Au_32_Br_8_[C_16_TA^+^•Br^-^]_12_ nanoclusters and matching 3D models at the same scale. Scale bars equal 1 nm.


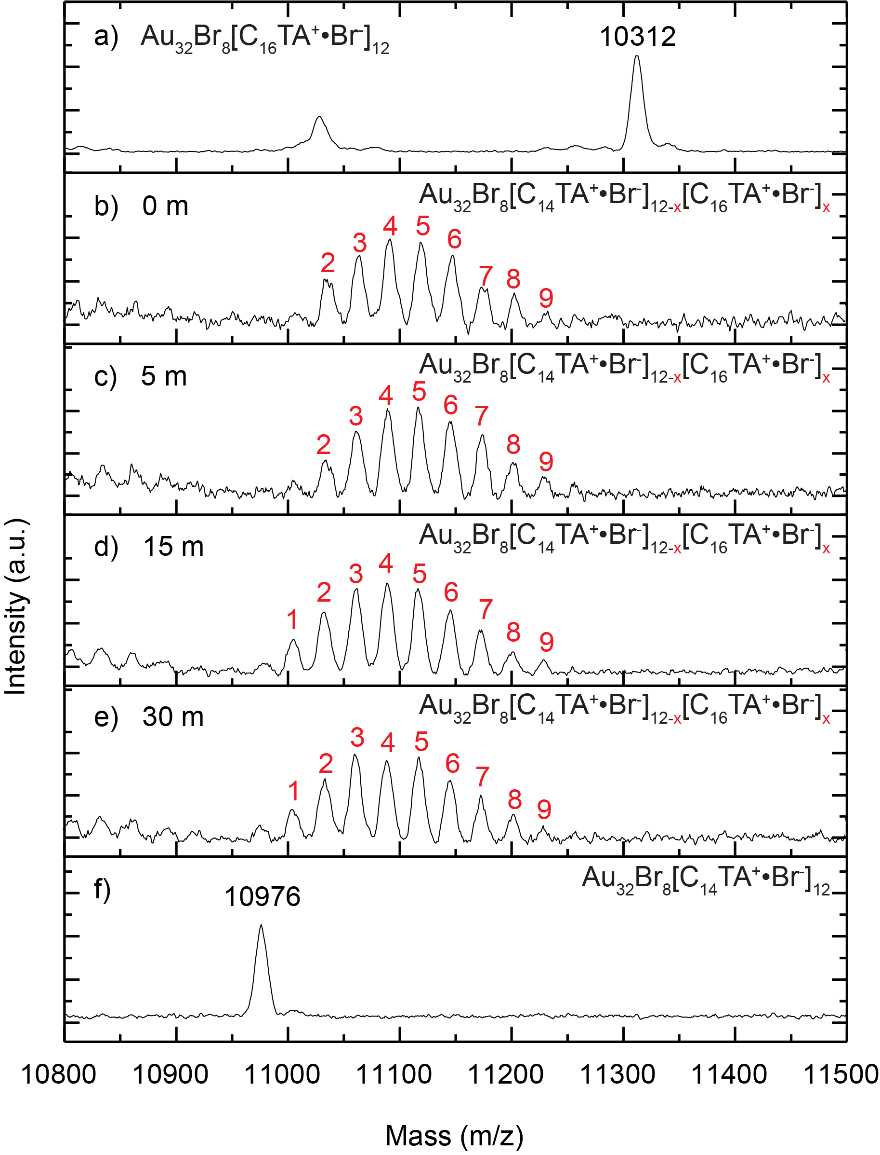


**f**

**e**

**d**

**c**

**b**

**a**

(*m*/*z*)

**Supplementary Figure 19.** Time-resolved ligand exchange in which Au_32_Br_8_[C_16_TA^+^•Br^-^]_12_ clusters are added to a solution containing 37.5 mM C_16_TAB and 12.5 mM C_14_TAB. **a** Au_32_Br_8_[C_16_TA^+^•Br^-^]_12_, **b**-**e** ligand exchange at 0, 5, 15, and 30 minutes, and **f** Au_32_Br_8_[C_14_TA^+^•Br^-^]_12_. *x* in the formula is labeled in red and placed over the peaks of mixed ligand clusters.


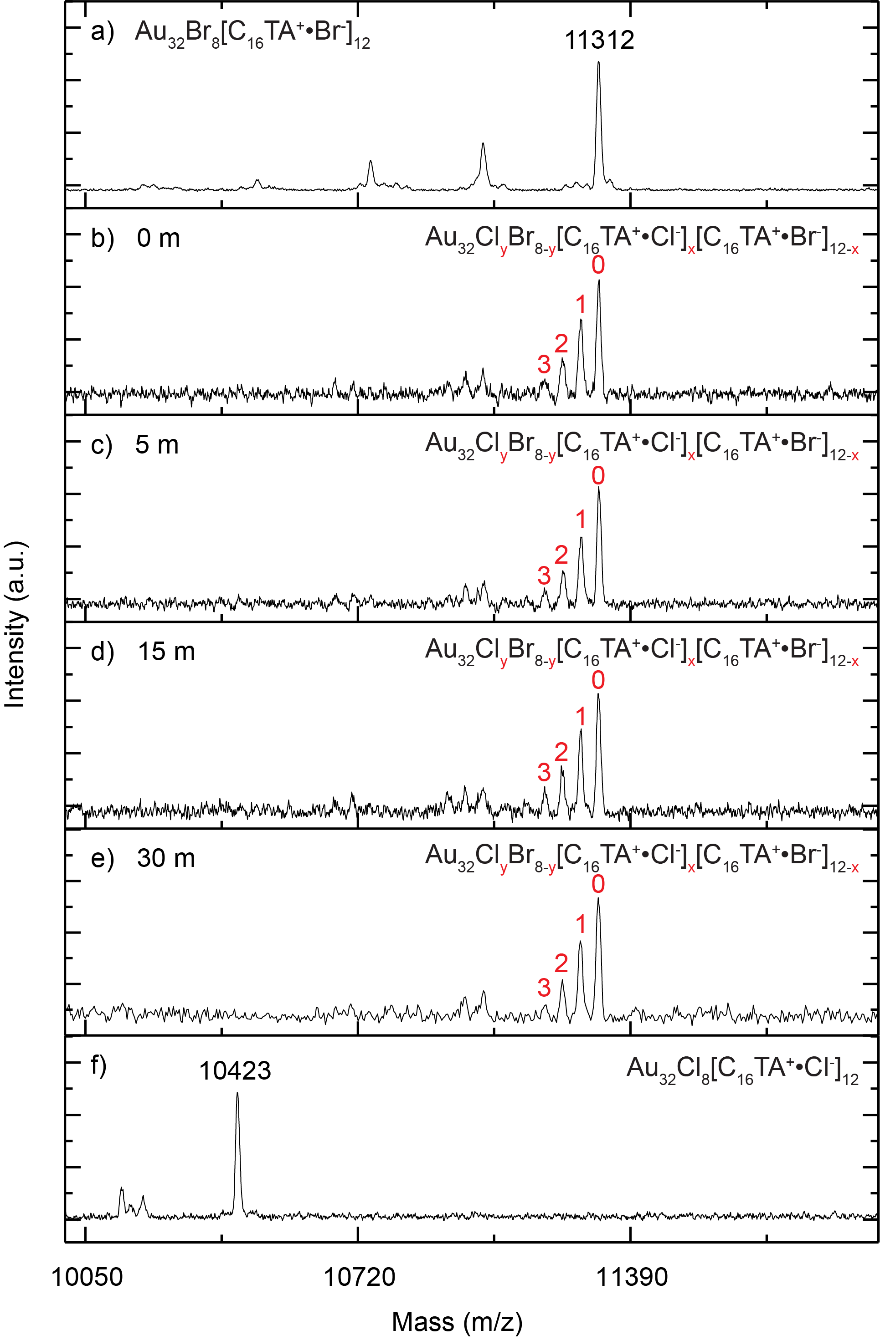


**f**

**a**

**b**

**c**

**d**

**e**

(*m*/*z*)

**Supplementary Figure 20.** Time-resolved ligand exchange in which Au_32_Br_8_[C_16_TA^+^•Br^-^]_12_ clusters are added to a solution of 25 mM C_16_TAB and 25 mM C_16_TAC. **a** Au_32_Br_8_[C_16_TA^+^•Br^-^]_12_, **b**-**e** ligand exchange at 0 m, 5 m, 15 m, 30 m, and **f** Au_32_Cl_8_[C_16_TA^+^•Cl^-^]_12_. *x*+*y* in the formula is labeled in red and placed over the peaks of hybrid clusters.


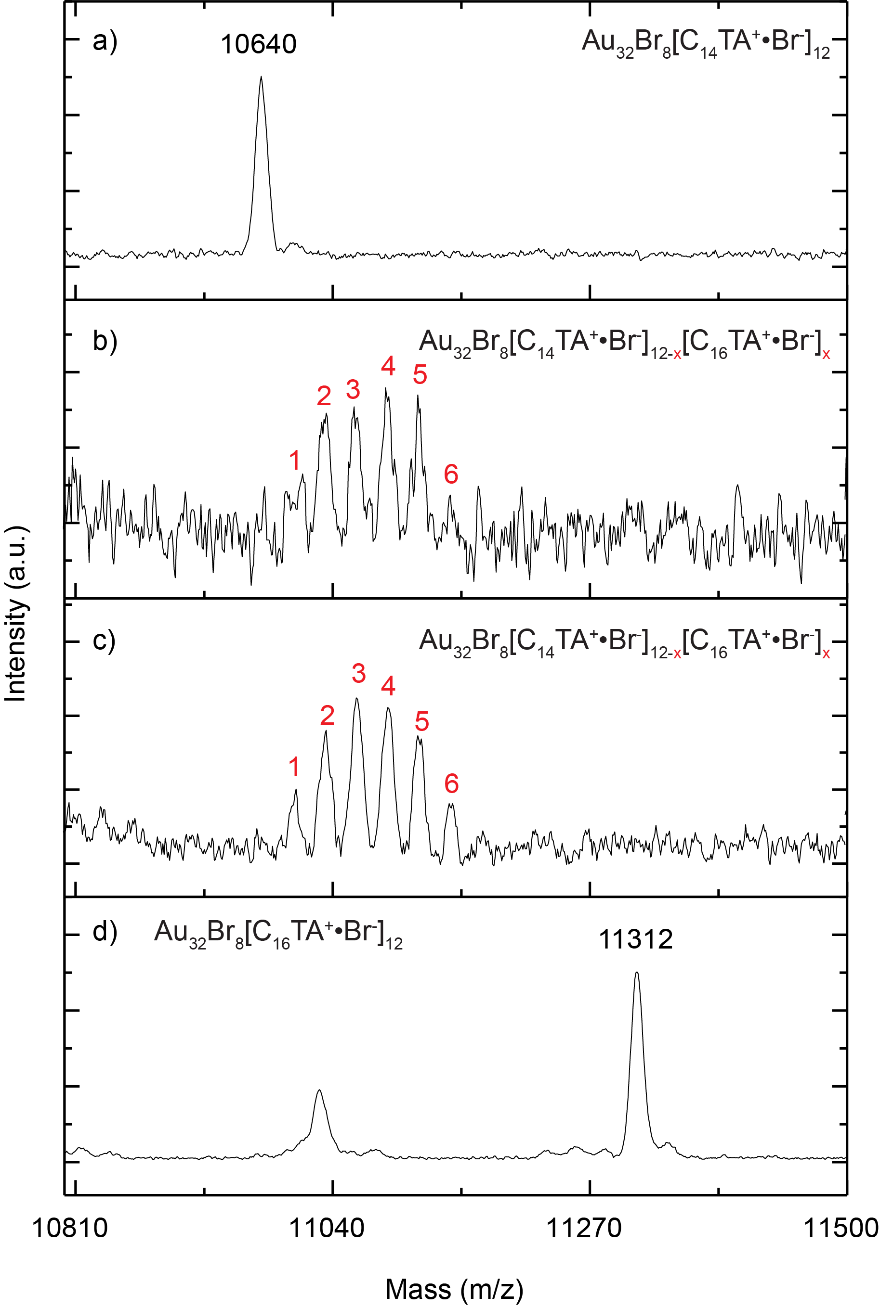


**b**

**c**

**d**

**a**

(*m*/*z*)

**Supplementary Figure 21.** Comparing the order in which ligand exchange reactions are conducted. **a** Au_32_Br_8_[C_14_TA^+^•Br^-^]_12_ synthesized in 50 mM C_14_TAB solution, **b** Au_32_Br_8_[C_14_TA^+^•Br^-^]_12_ cluster solution brought to 25 mM C_14_TAB and 25 mM C_16_TAB, **c** Au_32_Br_8_[C_16_TA^+^•Br^-^]_12_ cluster solution brought to 25 mM C_16_TAB solution and 25 mM C_14_TAB, **d** Au_32_Br_8_[C_16_TA^+^•Br^-^]_12_ synthesized in 50 mM C_16_TAB solution. Since panels **b** and **c** show similar distributions of mixed ligand clusters, we conclude both have reached the same equilibrium extent of exchange. *x* in the formula is labeled in red and placed over the peaks of mixed ligand clusters.

**b**

**a**


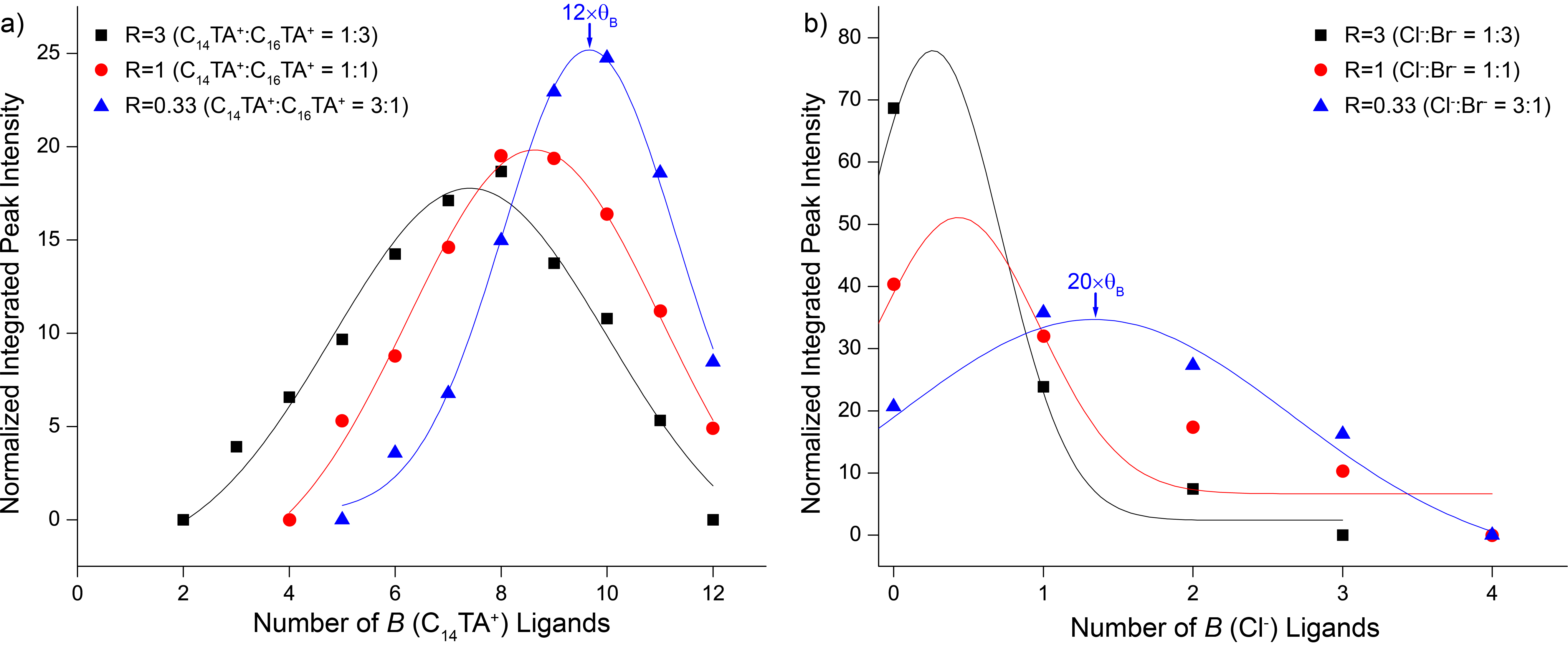


**Supplementary Figure 22.** Integrated peak intensities extracted from Figure 4b and d MALDI mass spectrometry data for C_n_TA^+^ **a** or X^-^ **b** ligand exchange. Values are normalized to 100 and plotted against the number of exchange events. Gaussian fits to these data allow for a value of *θ_B_* to be extracted for each sample with a different solution-phase ligand ratio, *R*. The *θ_B_* values determined from these fits were used in Supplementary Figure 23 to estimate ligand binding constant ratios.


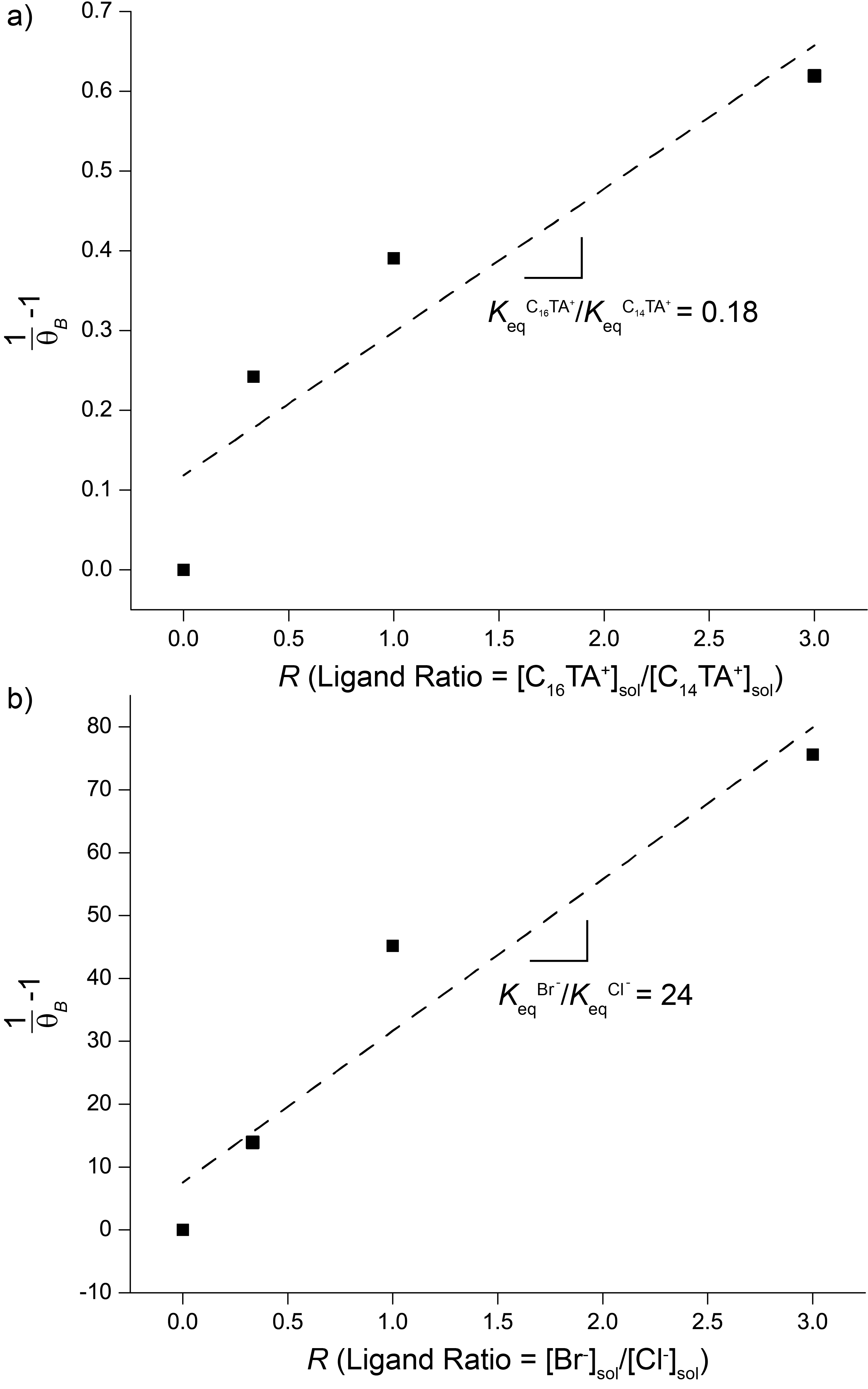


**b**

**a**

**Supplementary Figure 23.** Linear fits for two-component Langmuir isotherm analysis of **a** C_n_TA^+^ and **b** X^-^ ligand exchange. The slopes of these lines provide the ratio of equilibrium binding constants for the two ligands in solution. See methods for details.


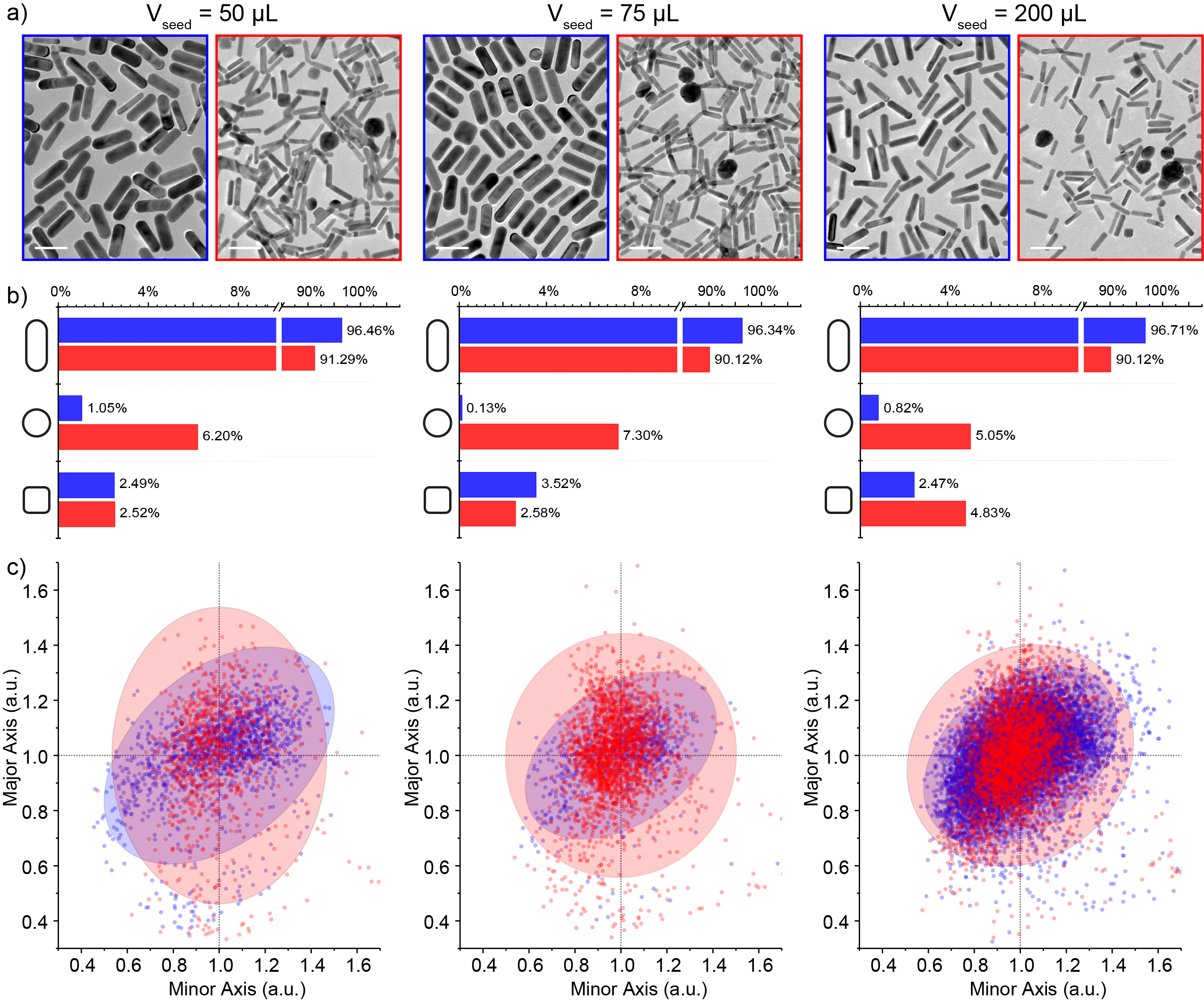


**b**

b)

**c**

c)

**a**

**Supplementary Figure 24.** Size distribution of gold nanorods synthesized with **a** 50 μL, **b** 75 μL, or **c** 200 μL Au_32_ nanocluster solution (blue) or traditional gold nanoparticle solution as seeds (red, see Methods for details). From top to bottom: representative TEM images, particle shape distribution, and nanorod size distribution with 95% confidence ellipse. Shown here are full datasets with the number of analyzed particles (*N*) being: *N* = 1525 and 7544 for Au_32_ and traditional seeds, respectively (50 μL sample, left); *N* = 766 and 2990 for Au_32_ and traditional seeds, respectively (75 μL sample, middle); and *N* = 11,172 and 4040 for Au_32_ and traditional seeds, respectively (200 μL sample, right). Data shown in main text Figure 5 is taken from the middle panel (**b**) with 500 datapoints selected at random to improve readability.


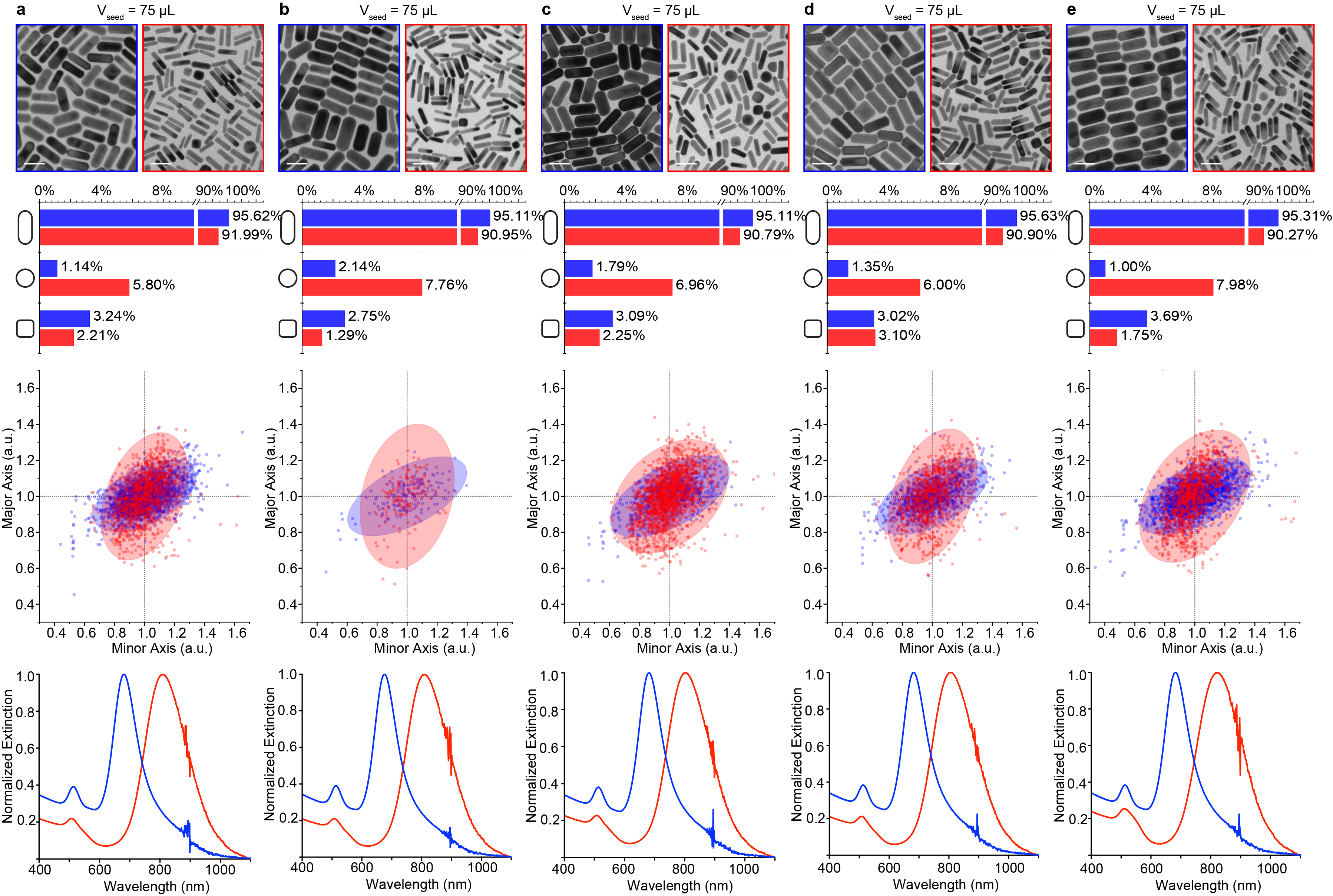


**Supplementary Figure 25.** Size distribution of gold nanorods synthesized with 75 μL of Au_32_ nanocluster solution (blue) or traditional gold nanoparticle solution as seeds (red) repeated 5 times (**a**-**e**) to test reproducibility. From top to bottom: representative TEM images, particle shape distribution, nanorod size distribution with 95% confidence ellipse, and normalized UV-vis spectra. The number of analyzed particles (*N*) are: *N* = 2505 and 1106 for Au_32_ and traditional seeds, respectively **a**; *N* = 161 and 154 for Au_32_ and traditional seeds, respectively **b**; *N* = 668 and 1925 for Au_32_ and traditional seeds, respectively **c**; *N* = 1201 and 823 for Au_32_ and traditional seeds, respectively **d**; *N* = 1951 and 1310 for Au_32_ and traditional seeds, respectively **e**. All replicates indicate that Au_32_-seeded samples contain a higher fraction of nanorod products and a narrower size distribution than their traditional seed counterparts.

**SUPPLEMENTARY References**

(1) Wang, Z. W.; Li, Z. Y.; Park, S. J.; Abdela, A.; Tang, D.; Palmer, R. E. Quantitative Z-contrast imaging in the scanning transmission electron microscope with size-selected clusters. *Phys. Rev. B* **2011,** *84*, 073408.

(2) Hartel, P.; Rose, H.; Dinges, C. Conditions and reasons for incoherent imaging in STEM. *Ultramicroscopy* **1996,** *63*, 93.

(3) Liao, Y., Practical Electron Microscopy and Database. 2nd ed.; https://www.globalsino.com/EM/, 2006.

(4) Williams, D. B.; Carter, C. B. *Transmission Electron Microscopy A Textbook for Materials Science*; Springer Science: New York, NY, 2009.

(5) Swenson, H.; Stadie, N. P. Langmuir’s Theory of Adsorption: A Centennial Review. *Langmuir* **2019,** *35*, 5409.

(6) Cao, Y.; Liu, T.; Chen, T.; Zhang, B.; Jiang, D.-e.; Xie, J. Revealing the etching process of water-soluble Au25 nanoclusters at the molecular level. *Nat. Commun.* **2021,** *12*, 3212.

(7) Yao, Q.; Yuan, X.; Fung, V.; Yu, Y.; Leong, D. T.; Jiang, D.-e.; Xie, J. Understanding seed-mediated growth of gold nanoclusters at molecular level. *Nat. Commun.* **2017,** *8*, 927.

(8) Humphrey, W.; Dalke, A.; Schulten, K. VMD: visual molecular dynamics. *Journal of molecular graphics* **1996,** *14*, 33.

(9) Price, D. J.; Brooks III, C. L. A modified TIP3P water potential for simulation with Ewald summation. *The Journal of chemical physics* **2004,** *121*, 10096.

(10) Te Velde, G. t.; Bickelhaupt, F. M.; Baerends, E. J.; Fonseca Guerra, C.; van Gisbergen, S. J.; Snijders, J. G.; Ziegler, T. Chemistry with ADF. *Journal of Computational Chemistry* **2001,** *22*, 931.

(11) Becke, A. D. Density-functional exchange-energy approximation with correct asymptotic behavior. *Phys. Rev. A: Gen. Phys.* **1988,** *38*, 3098.

(12) Perdew, J. P. Density-functional approximation for the correlation energy of the inhomogeneous electron gas. *Physical Review B* **1986,** *33*, 8822.

(13) Van Lenthe, E.; Baerends, E. J. Optimized Slater‐type basis sets for the elements 1–118. *Journal of computational chemistry* **2003,** *24*, 1142.

(14) Lenthe, E. v.; Baerends, E.-J.; Snijders, J. G. Relativistic regular two‐component Hamiltonians. *The Journal of chemical physics* **1993,** *99*, 4597.

(15) van Lenthe, E.; Baerends, E.-J.; Snijders, J. G. Relativistic total energy using regular approximations. *The Journal of chemical physics* **1994,** *101*, 9783.

(16) Van Lenthe, E.; Ehlers, A.; Baerends, E.-J. Geometry optimizations in the zero order regular approximation for relativistic effects. *The Journal of chemical physics* **1999,** *110*, 8943.

(17) Dass, A.; Stevenson, A.; Dubay, G. R.; Tracy, J. B.; Murray, R. W. Nanoparticle MALDI-TOF Mass Spectrometry without Fragmentation: Au25(SCH2CH2Ph)18 and Mixed Monolayer Au25(SCH2CH2Ph)18-x(L)x. *J. Am. Chem. Soc.* **2008,** *130*, 5940.

(18) Kile, D. E.; Chiou, C. T. Water solubility enhancements of DDT and trichlorobenzene by some surfactants below and above the critical micelle concentration. *Environmental Science & Technology* **1989,** *23*, 832.
